# Phylodynamics and Human-Mediated Dispersal of a Zoonotic Virus

**DOI:** 10.1371/journal.ppat.1001166

**Published:** 2010-10-28

**Authors:** Chiraz Talbi, Philippe Lemey, Marc A. Suchard, Elbia Abdelatif, Mehdi Elharrak, Nourlil Jalal, Abdellah Faouzi, Juan E. Echevarría, Sonia Vazquez Morón, Andrew Rambaut, Nicholas Campiz, Andrew J. Tatem, Edward C. Holmes, Hervé Bourhy

**Affiliations:** 1 Institut Pasteur, Unit Lyssavirus Dynamics and Host Adaptation, WHO Collaborating Centre for Reference and Research on Rabies, Paris, France; 2 Rega Institute, Katholieke Universiteit Leuven, Leuven, Belgium; 3 Departments of Biomathematics and Human Genetics, David Geffen School of Medicine, and Department of Biostatistics, School of Public Health, University of California, Los Angeles, Los Angeles, California, United States of America; 4 Institut Pasteur d'Algérie, Laboratoire de la Rage, Recherche et Diagnostic, Alger, Algérie; 5 Biopharma Laboratoire, Rabbat, Maroc; 6 Institut Pasteur du Maroc, Laboratoire de Virologie Médicale, Casablanca, Maroc; 7 Instituto de Salud Carlos III, Servicio de Microbiología Diagnóstica, Madrid, Spain; 8 Institute of Evolutionary Biology, University of Edinburgh, Ashworth Laboratories, Edinburgh, United Kingdom; 9 Fogarty International Center, National Institutes of Health, Bethesda, Maryland, United States of America; 10 Department of Geography, University of Florida, Gainesville, Florida, United States of America; 11 Emerging Pathogens Institute, University of Florida, Gainesville, Florida, United States of America; 12 Center for Infectious Disease Dynamics, Department of Biology, The Pennsylvania State University, University Park, Pennsylvania, United States of America; Fred Hutchinson Cancer Research Center, United States of America

## Abstract

Understanding the role of humans in the dispersal of predominately animal pathogens is essential for their control. We used newly developed Bayesian phylogeographic methods to unravel the dynamics and determinants of the spread of dog rabies virus (RABV) in North Africa. Each of the countries studied exhibited largely disconnected spatial dynamics with major geo-political boundaries acting as barriers to gene flow. Road distances proved to be better predictors of the movement of dog RABV than accessibility or raw geographical distance, with occasional long distance and rapid spread within each of these countries. Using simulations that bridge phylodynamics and spatial epidemiology, we demonstrate that the contemporary viral distribution extends beyond that expected for RABV transmission in African dog populations. These results are strongly supportive of human-mediated dispersal, and demonstrate how an integrated phylogeographic approach will turn viral genetic data into a powerful asset for characterizing, predicting, and potentially controlling the spatial spread of pathogens.

## Author Summary

At least 15 million doses of anti-rabies post-exposure prophylaxis are administered annually worldwide, and an estimated 55,000 people die of rabies every year. Over 99% of these deaths occur in developing countries, predominantly in Asia and in Africa where rabies is endemic in domestic dogs. Despite the global health burden due to rabies, little is known about the patterns of the spread of dog rabies in these endemic regions. We used recently developed Bayesian analytical methods to unravel the dynamics and determinants of the spatial diffusion of dog rabies viruses in North Africa based on viral genetic data. Our analysis reveals a combination of restricted spread across administrative borders, the occasional long-distance movement of rabies viruses, and a strong fit between spatial spread of the virus and road distances between localities. Together, these data indicate that by transporting dogs, humans have played a key role in the dispersal of a major animal pathogen. Our studies therefore provide essential new information on the transmission dynamics of rabies in Africa, and in doing so will greatly assist in future intervention strategies.

## Introduction

Every year approximately 55,000 people die from rabies [1]. Over 99% of these deaths occur in developing countries where rabies virus (RABV; negative-sense RNA virus, family *Rhabdoviridae*) is endemic in the domestic dog [2]. Rabies has been neglected across much of Asia and Africa, despite becoming an increasing problem in the recent decades [1], [3], [4]. Although the history of rabies in Africa prior to the 20th century is uncertain [5], the currently circulating dog rabies virus is thought to have emerged during the 19th and 20th centuries [6], [7]. Despite the importance of dogs as vectors for human rabies, little is known about the spatial and temporal dynamics of rabies in this major reservoir species, or the processes responsible for its maintenance in specific geographic localities. In particular, the role of human activities in mediating the spread of dog RABV is unclear, nor is it known how landscape characteristics, including human infrastructures such as roads, affect RABV dispersal within dog populations. However, such information is critical to revealing the determinants of RABV transmission and hence for its control in the domestic dog.

We used a recently developed probabilistic approach [8] to determine the spatial and temporal dynamics of dog RABV transmission from a large-scale gene sequence study. We encode different phylogeographic scenarios of viral spread, as well as different landscape features, in a model-based approach, and choose among these models in a quantitatively rigorous fashion. Our focus was on dog populations in North Africa where RABV has been endemic for more than a century, and our key aim was to determine how ecological, anthropogenic and evolutionary dynamics shape the spatial distribution and spread of this important zoonotic pathogen [9], [10], [11], [12].

## Results/Discussion

**Figure 1:**
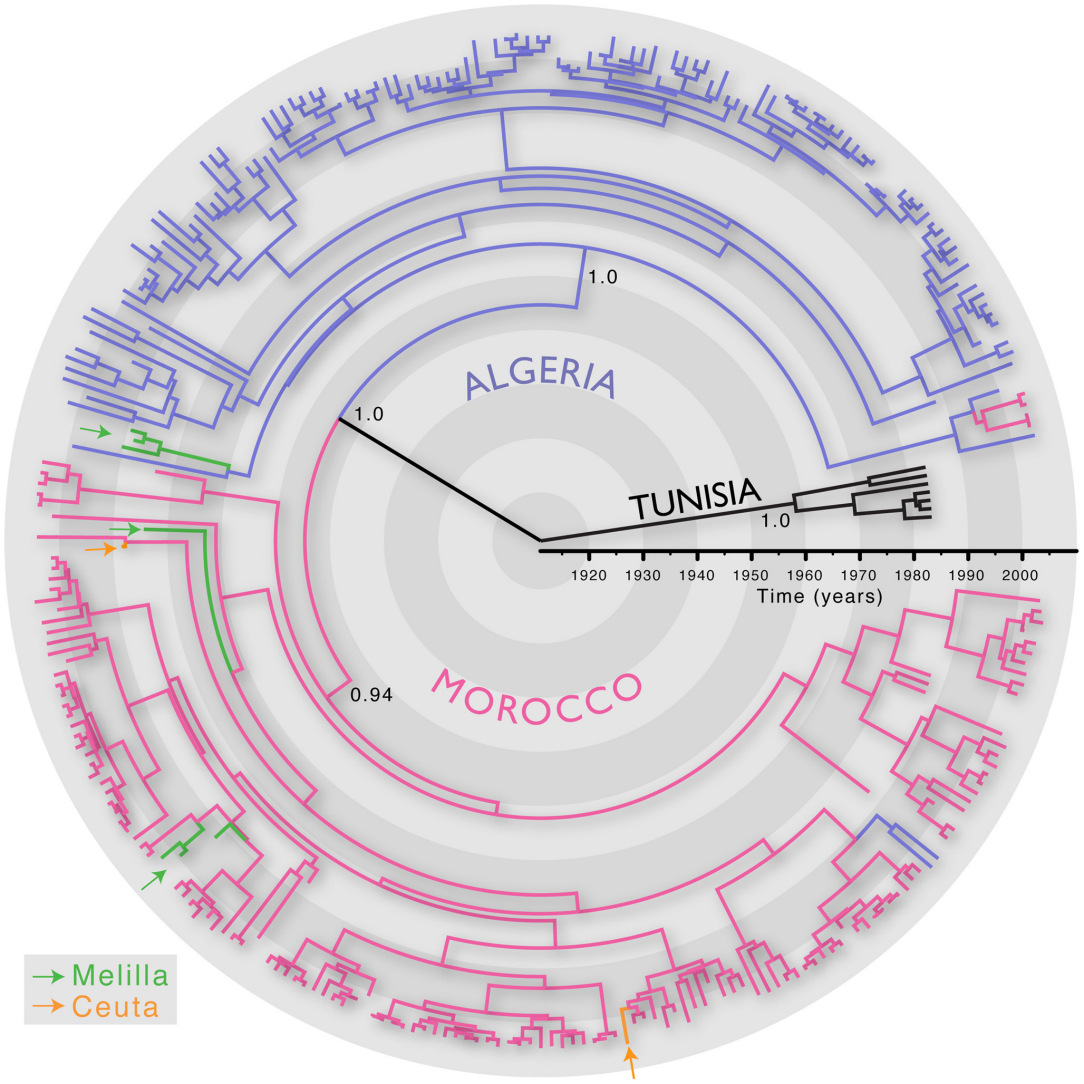
MCC tree of 287 sequences of the Africa 1 clade, estimated from the N, P and G-L genes and intergenic regions of dog RABV, and showing the spatial structure of the viral lineages. Branches are colored according to the locations of viral sampling. Branch lengths are estimated in time units (years) as indicated by the time bar. Posterior clade probability values (>0.9) are shown for key nodes. Although Melilla and Ceuta are considered Spanish locations they are in fact Moroccan enclaves. As such they do not represent virus dispersal across the strait of Gibraltar.

We first inferred the evolutionary history of 287 RABV sequences (3080 nt; encompassing the whole N, P and intergenic G-L region) sampled from Algeria, Morocco, Tunisia and the Spanish territories from North Africa (Ceuta and Melilla) between 1986 and 2008. All these viruses are assigned to the Africa 1 genotype (relevant epidemiological information for all RABV isolates analysed in this study is presented in Table S1 in Supporting Information S1). We estimated the timescale of this evolutionary history using a Bayesian Markov chain Monte Carlo (MCMC) approach [13]. The most recent common ancestor of all the North African RABV sampled here was estimated to have existed between 1878–1945, supporting previous suggestions that dog RABV was periodically responsible for local sporadic epidemics in the middle of the 19th century [14], and that rabies became enzootic in this entire region during the 20th century. More generally, this timescale is consistent with the expanding European colonial influence in North Africa [7]. This analysis also revealed distinct phylogenetic lineages in Algeria, Morocco and Tunisia, indicating that viruses generally grouped according to their country of origin (Figure 1). This result is unexpected if the virus is only dispersed through the local movement of animals as observed in wildlife rabies [9], [10] as these would not respect geo-political boundaries. Indeed, we found only a few exceptions to the country-specific clustering, such as two Algerian sequences within the Moroccan clade and four Moroccan sequences in the Algerian clade. The Africa 1 clade is therefore consistent with the general phylogeographic pattern observed for dog RABV at reasonably large geographic scales; a series of spatially distinct clusters that experience relatively little contact among them [6], [7].

**Figure 2:**
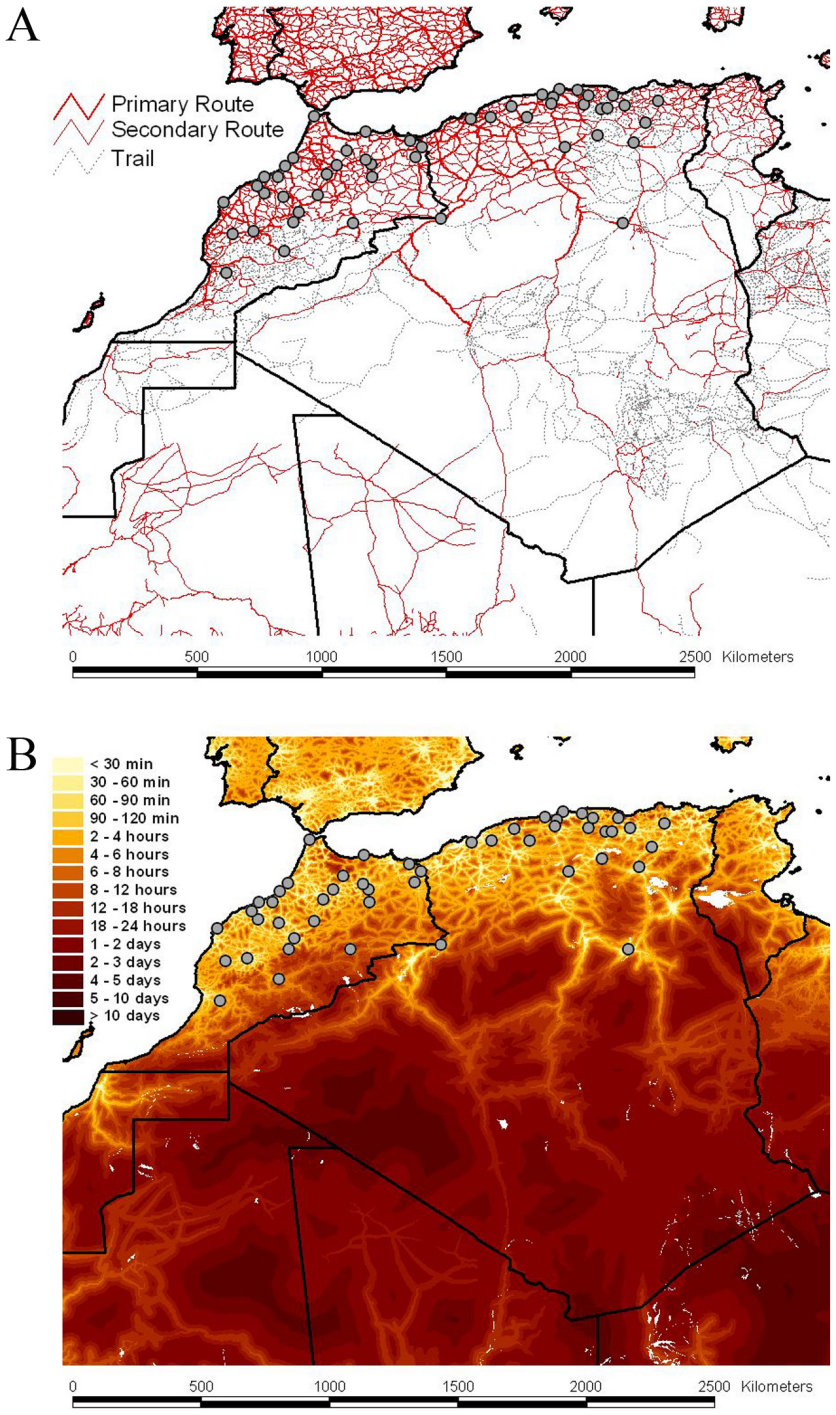
Road network, accessibility and sampling locations of RABV samples. The road network and the accessibility are shown in panels A and B, respectively The border between the countries is indicated by dark lines. Specific locations were unavailable for Tunisian samples. The color gradient indicates the estimated travel time to the nearest city of population greater than 50,000 people, with yellow at one extreme indicating low travel times (<30 min) and red at the other extreme indicating long travel times (>10 days). The data are available for download from http://gem.jrc.ec.europa.eu/.

To analyze intra-country patterns of viral transmission in more detail, we considered a stochastic diffusion process among the 20 (Algeria) and 28 (Morocco) sampling localities for which most data were available (Figure 2 and Figure S1 in Supporting Information S1). We quantified the degree of spatial admixture using a modified Association Index (AI, [8], [15]), and by summarizing the number of inferred transitions to each location within Algeria and Morocco (Table 1) based on an analysis in which rates of diffusion between each pair of locations were estimated. Although these analyses reveal that there is still significant spatial structure within each country (p<0.001), the AIs are considerably higher (0.67 [0.62–0.73] and 0.55 [0.51–0.63] for Algeria and Morocco, respectively) than those found for rabies at a larger spatial scale (e.g., 0.087 [0.043–0.132] for the Africa 2 lineage in Central and West Africa) [8], indicating weaker spatial structure at the within-country level. The summaries of transitions to each location generally identify multiple independent introductions of viruses in each location from which several samples were obtained (Table 1). Overall, the number of independent transitions to densely sampled locations is lower in Morocco than Algeria, in agreement with the lower AI for Morocco. Taken together with the strong spatial structure across countries, these results suggest that a relatively fluid RABV diffusion process within countries is restricted by geopolitical boundaries at larger scales.

**Table 1:** Transition summaries to each location in Algeria and Morocco.

Algeria		Morocco	
Location (# samples)	Transitions*	Location (# samples)	Transitions*
Ain Defla (3)	1 (1–3)	Agadir (8)	2 (1–3)
Alger (26)	9 (6–14)	Al Hoceima (2)	2 (2–3)
Batna (1)	1 (1–2)	Azilal (2)	1 (1–2)
Bejaia (5)	5 (4–7)	Beni Mellal (3)	1 (1–3)
Biskra (1)	1 (1–3)	Benslimane (9)	8 (6–9)
Blida (15)	12 (8–15)	Berkane (6)	2 (1–5)
Borj Bou Arreridj (3)	3 (1–4)	Casa (8)	5 (3–7)
Bouira (6)	4 (2–6)	Chichaoua (3)	3 (3–4)
Boumerdes (11)	9 (6–11)	El Hajeb (2)	2 (2–3)
Chlef (5)	1 (1–3)	Errachidia (4)	1 (1–2)
Constantine (1)	1 (1–2)	Fes (1)	1 (1–2)
Djelfa (2)	2 (2–4)	Figuig (2)	2 (2–3)
Medea (8)	7 (6–9)	Ifrane (1)	1 (1–2)
Msila (6)	4 (3–6)	Jerrada (1)	1 (1–2)
Ouargla (1)	1 (1–3)	Kenitra (3)	3 (3–4)
Relizane (2)	2 (2–4)	Khenifra (2)	1 (1–2)
Setif (1)	1 (1–3)	Khouribga (1)	1 (1–2)
Tipaza (13)	9 (5–11)	Marrakech (22)	2 (2–3)
Tissemsilt (3)	3 (2–4)	Nador (2)	2 (2–3)
Tizi-Ouzou (4)	4 (2–5)	Ouarzazate (7)	2 (2–3)
		Oujda (5)	2 (1–5)
		Rabat (7)	6 (6–7)
		Settat (4)	3 (2–4)
		Sidi Kacem (9)	6 (5–8)
		Tanger (11)	1 (1–6)
		Taounate (1)	1 (1–2)
		Taourirt (3)	3 (3–4)
		Taza (2)	2 (2–3)

*Transitions to each location are summarized based on the node location realizations in each tree of the posterior distribution. We list the modal number of transitions and the HPD intervals. Note that location transitions can occur on relatively deep branches in the tree, sometimes even resulting in transitions exceeding the number of sequences for a particular location.

To identify the factors that may explain RABV spread, we incorporated several potential predictors as relative diffusion rates among each pair of locations, and tested these against equal rates of diffusion. Specifically, we considered geographical distances (great-circle distances), human population size, road distances and spatial accessibility measures. Road distances were derived from transport network data (Figure 2) and demonstrated a strong correlation with great-circle distances (*r* = 0.96). More detailed landscape features, which may imply multiple, direct and indirect pathways connecting the different localities sampled in this study, were represented by accessibility data. These data reflect the travel time to the nearest major city using road/track-based travel [16] and were less correlated with geographical distances (*r* = 0.61). We employed circuit theory to translate the accessibility landscape into an origin-destination distance matrix (the so-called ‘isolation by resistance model’) [17]. We also tested a simple gravity model of viral spread that, in the absence of real dog population sizes for the locations involved, was based on human population sizes for the discrete as a proxy. Finally, we also used population sizes in a landscape approach, similar to accessibility measures, to construct a population surface matrix [18].

Marginal likelihood estimates of the model fit of these different predictors suggested that RABV spatial dynamics are best described by road distances (Table 2). This was consistent across both countries and again supports human-assisted dispersal of rabies-infected dogs. As expected by their high correlation, geographical distances provided only a marginally lower fit compared to road distances. Only the population surface provided inconsistent results between both countries; whereas this model competes with road distances in Algeria, the population surface did not provide a good fit to the Moroccan data (Figure S2 in Supporting Information S1). Although accessibility did not seem to explain RABV diffusion as well, we note that all samples were obtained from relatively accessible parts of Morocco and Algeria.

**Table 2:** Marginal (log) likelihood estimates for the fit of different phylogeographic diffusion predictors in Algeria and Morocco.

	Algeria	Morocco
Equal rates	−295.2	−320.0
Great circle distances	−273.0	−299.8
Population sizes*	−330.3	−381.0
Gravity model**	−342.1	−388.6
Population surface	−272.6	−335.9
**Road distances**	**−272.1**	**−298.5**
Accessibility	−289.4	−313.7

*diffusion rates among each pair of locations were fixed to the product of the populations sizes of the locations involved.

**In the gravity model, rates were fixed to the product of the populations sizes divided by the great circle distance between the locations involved. The best fitting model and associated marginal log likelihoods are listed in bold. Marginal log likelihoods for analyses in which rates were estimated from the data (−276.5 and −301.3 for Algeria and Morocco, respectively) indicate that the best fitting models with fixed rate parameters compete with far more flexible, but very parameter-rich models.

To quantify and compare the dissemination process with previous results, we estimated the rate of RABV gene flow among the sampled isolates using ‘Markov jump’ counts [19] of location state transitions and their reward-associated distances between locations across each branch. The posterior average rate of viral gene flow among localities estimated for Algeria was 26 km/yr (95% highest probability density interval: 18–34) and 33 (23–43) km/yr based on great circle distances and road distances, respectively. Somewhat higher viral gene flow rate estimates were obtained for Morocco with 42 (26–58) km/yr and 51 (34–72) km/yr for great circle distances and road distances, respectively. We note that the rates of viral gene flow estimated here are highly dependent of the scale of sampling such that comparison may only prove useful at the same geographic scale. However, these estimates were 2.7 to 4.4 times higher than those recorded in established enzootic situations in wildlife animals [9], again suggestive of human-mediated transmission. Although it is theoretically possible that these relatively high rates reflect epidemic waves periodically moving through this geographical region [20], particularly since similar rates have been observed in wild carnivores during epidemic spread [9], [10], such waves were not observed in the geographical areas studied here and where the virus appears to be largely enzootic.

**Figure 3:**
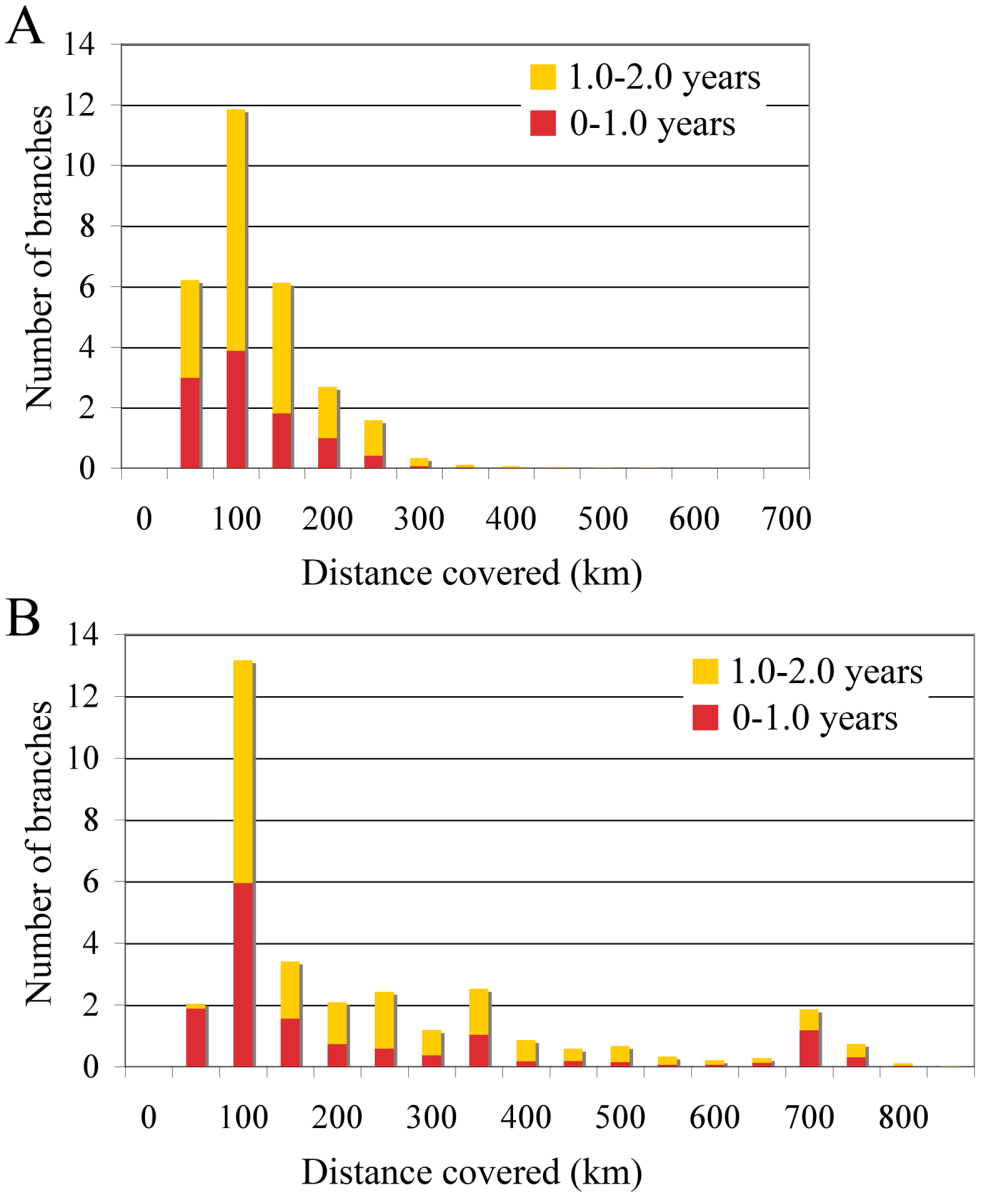
Short-time virus gene flow events in Algeria and Morocco. Branches that harbor an expected state change (different location at parent and daughter node) were binned according to the distance covered along that branch in Algeria (A) and Morocco (B). This was carried out separately for branches along which state changes occurred in less than 1 year, and between 1 and 2 years.

The occasional mixing of sequences from different locations at the tips of the inferred tree (Figure 1) is suggestive of long distance spread in relatively little time (6 months to one year). To quantify such rapid and long distance spread, we summarized the posterior distribution of distances covered along individual branches (Figure 3). We focused on branches along which inferred location state changes occurred in a time period of less than 1 year, and between 1 and 2 years. As a control, we analysed the branches without inferred state changes; as expected these all had negligible Markov jump count distances (not shown). Across the posterior distribution of trees, we observed between 5 and 13 branches per tree that have a time length less than 2 years and cover a distance of more than 200 km, and 12 to 13 branches that cover a distance of about 100 km (Figure 3). Importantly, our ability to clearly detect long-distance movement is limited to branches representing short evolutionary times; longer branches could also harbour such events, providing an explanation for the relatively high average rates of viral gene flow.

**Figure 4:**
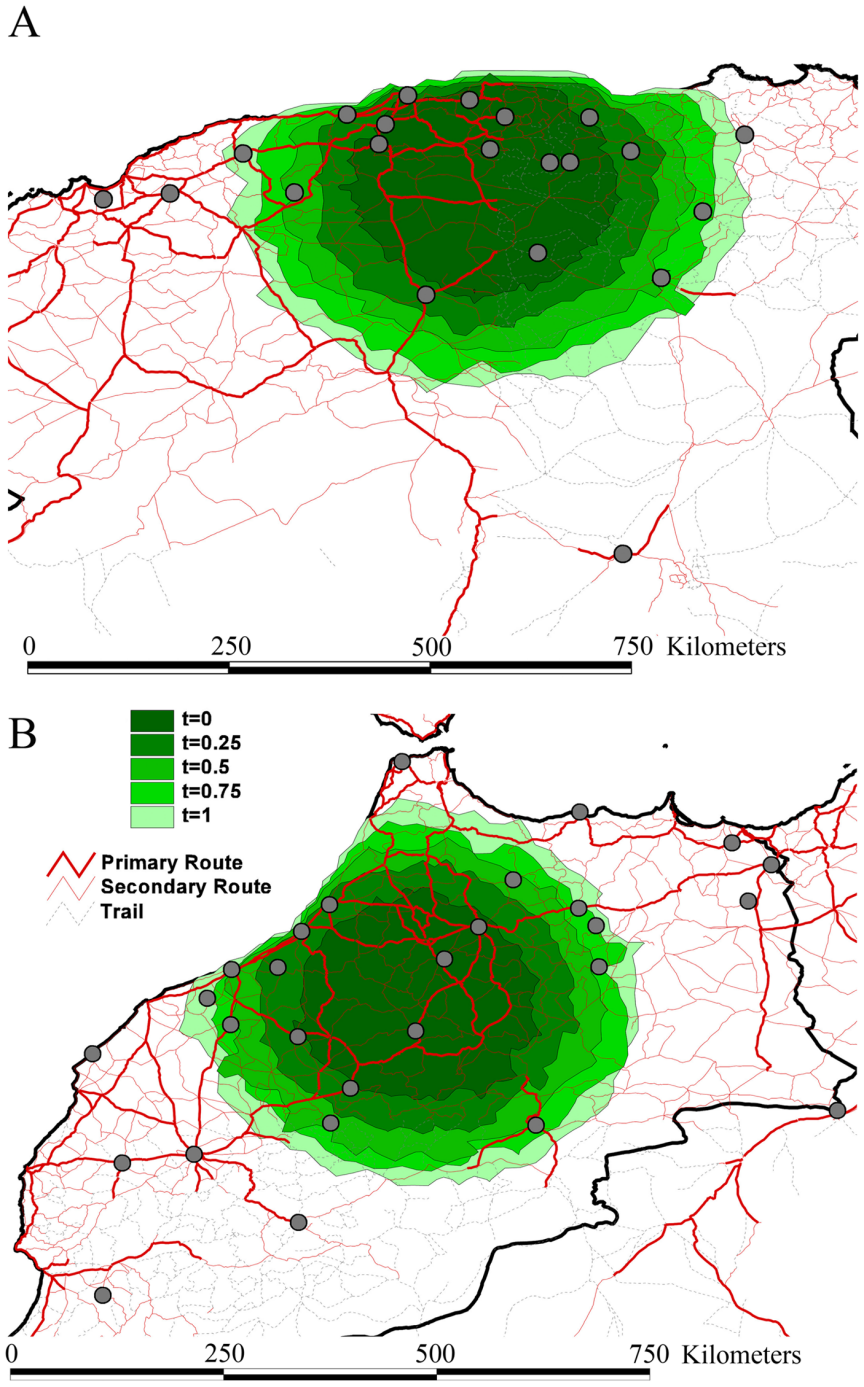
Spatial simulation based on the inferred evolutionary histories and epidemiological parameters of rabies spread in Algeria (A) and Morocco (B). We report the simulated spread using contours represented under the form of polygons. Different contours represent different simulation scenarios: for time = 0.25 to time = 1.0, we force the virus to move consistently in the same direction for successive transmissions during a time period of three months to one year. The dark green contour in the centre represents simulated spread with each infection taking a new and random direction, while the increasingly lighter ones represent time = 0.25 to time = 1.0.

The rates of viral gene flow we estimate among the sampled isolates from Algeria and Morocco contrast with those of spatial RABV movement in an African dog population that should experience very limited human-mediated dissemination of rabid dogs [21]. In this case, the spatial dispersal of single RABV infections was estimated to be predominantly less than 2 km (and always smaller than 20 km). Considering that the average incubation period of RABV is between 22 to 29 days [20], [21], [22], [23], [24], it is clear that such long distances as those recorded in our study could only be achieved with at least some human intervention. To investigate more formally how the RABV distribution we observe in Algeria and Morocco contrasts with the patterns of spread we would expect from transmission dynamics in African dogs alone (i.e. without human intervention), we simulated a phylogeodynamic process based on epidemiological parameters obtained from detailed analyses of rabies transmission biology [20], [21]. In particular, we considered epidemiologically informed virus movement over all evolutionary histories in the posterior distribution resulting from our phylodynamic inference (see Supplementary Information). In our spatial simulation we analyze cases in which (i) each new infection takes a random direction in continuous space, or (ii) subsequent infections consistently take the same direction (Figure 4). Although the latter may not be very realistic, it should resemble virus movement along roads. For both Algeria and Morocco, spatial diffusion is initiated at the centre of the sampling locations, such that the process has the largest probability to cover these locations *a priori*. When assuming up to one year of movement these simulations clearly show that RABV could not have spread to the same extent as shown by the current sampling in Algeria and Morocco if the virus was simply being transmitted by dog dispersal alone. Even if we enforce a year of successive RABV transmissions in the same direction, which is highly implausible given the observed dynamics of dog RABV in a local setting [21], the simulations still do not attain the observed spatial RABV spread. In addition, the distances realized by dispersal in random directions along branches less than 2 years were all less than 60 km, which is far more restricted than estimated for the real data (Figure 3).

Rabies is a prime example of an infectious disease in which dispersal can be exacerbated by animal movement mediated by humans. This is illustrated by raccoon rabies in Virginia, USA [25], dog rabies in Indonesia in Flores Island [26], in Bali (F.X. Meslin, Personal communication) and in parts of Europe [27]. Each epidemic resulted in enormous expenditure on rabies post exposure prophylaxis in humans and animal vaccination programs [28], [29], [30], [31]. Importantly, our study allows us to quantify rates of viral gene flow among sampled dog isolates (between 18 and 72 km/yr) in a mixed geographic and socio-economic landscape, such as those characterized by Algeria and Morocco where there is currently little dog vaccination. In addition, our analysis suggests that the human-mediated dispersal of infected dogs is likely to continue to play a major role in the transmission of RABV in geographical areas where it has been present for many years. Indeed, our observations of administrative borders that restrict a relatively fluid pattern of spread, the occasional long-distance movement of viruses to particular countries, and the fit between spatial dynamics and road distances, all point to the displacement of rabies-infected dogs by humans.

Understanding the frequency and distance of movements of potentially infected animals is of paramount importance in predicting the spread of viral infections [32], [33]. In addition, such information has important implications for disease control; understanding the conditions under which the containment of wildlife [34] and dog rabies can reliably be achieved will assist in the long term goal of eliminating animal RABV. In particular, that humans mediate the transmission of RABV among dogs in North Africa requires that intervention procedures are implemented more rapidly than in situations in which humans play little or no role in viral transmission. The high cost associated with surveillance underscores the importance of sampling design and the development of cost-effective monitoring and testing approaches [9], [12], [35]. In addition, this study illustrates the power of phylogeographic approaches [8] to identify the factors responsible for the spread of major animal and zoonotic pathogens. By integrating spatial dynamics with temporal inferences, the Bayesian analysis utilized here constitutes a powerful new tool that may complement traditional epidemiological methods in studying the effects of human behaviour on the evolution of zoonotic viruses.

## Materials and Methods

### Data selection

The Office of Veterinary Public Health Services of the different countries coordinates follow-up per animal bites. The respective state health departments (in conjunction with qualified laboratories that they designate) conduct all collection, observations, and euthanization (if necessary) of animals suspected of rabies, according to established national standardized protocols. A total of 287 isolates sampled from Morocco, Algeria, Tunisia and Spain (Ceuta and Melilla) were collected by the authors within the framework of these qualified laboratories, and sequenced. These samples were collected from dead animals suspected of rabies so that submission for laboratory rabies diagnosis is mandatory. Spatial co-ordinates and time of sampling, covering a period of 22 years (1986–2007), were available for the majority of these isolates. Relevant epidemiological information and GenBank accession numbers for all RABV isolates analysed in this study are presented in Table S1 in Supporting Information S1. All the locations sampled in this study experienced cases every year. As such, our sampling does not focus on areas that have been free of rabies in the recent past.

To perform a reliable evolutionary analysis of dog RABV circulating in North Africa, we aimed at selecting a genetic region with sufficient phylogenetic information. To this end, we sequenced a total of 3080 nt encompassing the N, P and intergenic G-L region. Total RNA from the original brain samples was extracted using Trizol reagent (Invitrogen) according to the manufacturer's instructions. RT-PCRs and sequencing reactions were performed as described previously [6], [36]. All sequences obtained have been deposited in GenBank (accession numbers GU798102–GU798962).

Additional primers used in this study were N1280 (5′- AGTCAGTTCTAATCATCAAGC-3′), M138(5′-AAGTTCCTYATGTTYTTCTTGC-3′), G(5′GACTTGGGTCTCCCGAACTGGGG-3′) and L (5′-CAA AGG AGA GTT GAG ATT GTA GTC-3′), at positions 1348–1368, 2632–2653, 4666–4688 and 5512–5535, respectively, of the lyssavirus genome [37].

Multiple sequence alignment was performed using MUSCLE available through the Muscle web interface (http://www.ebi.ac.uk/Tools/muscle/index.html) [38]. All alignments are available from the authors on request.

### Bayesian evolutionary analyses of spatiotemporal RABV dynamics

To investigate the evolutionary relationships and time to common ancestry among RABV lineages circulating in north Africa, we reconstructed the phylogenetic history for the entire data set using Bayesian Markov chain Monte Carlo (MCMC) analysis implemented in the BEAST package [13]. BEAST incorporates sampling time information to estimate evolutionary rates and a posterior distribution of time-scaled trees. We employed a GTR model of nucleotide substitution with gamma-distributed rate variation among sites and a relaxed (uncorrelated log-normal) molecular clock model [39]. We specified a Bayesian skyline plot model as flexible tree prior [40]. All chains were run for a sufficient length and convergence was diagnosed using Tracer (http://tree.bio.ed.ac.uk/software/tracer/) ignoring 10% of the chain as burn-in. Evolutionary history was summarized using an annotated Maximum Clade Credibility (MCC) phylogenetic tree. Posterior probability values provide an assessment of the degree of support for each node on the tree.

To reconstruct the spatial dynamics of dog-associated RABV spread and investigate the role of different diffusion predictors in shaping the epidemic in both Morocco and Algeria, we extracted two data sets with their specific spatial and temporal co-ordinates: (i) A total of 117 Algerian sequences (3080 nt) collected from dogs in 20 cities over 7 years (from 2001 to 2008), and (ii) a total of 133 Moroccan sequences (3080 nt) sampled from dogs in 28 cities between 2004 and 2008. For all these isolates precise dates (month) of sampling and geographical localities (city) are available (Table S2 in Supporting Information S1).

As an additional component in the fully probabilitistic Bayesian inference framework, we consider a discretized diffusion process among the sampling locations in both countries, formalized as a continuous time Markov chain (CTMC) model [8]. A CTMC is fully characterized using a matrix that describes the rate of movement from location state *i* to *j* for every pair of locations. To efficiently estimate the diffusion process from a single observation (a single location realization for each sequence), we restrict the parameterization to a sparse set of rates that adequately explains the phylogeographic dispersal process using Bayesian Stochastic search variable selection (BSSVS). This BSSVS procedure also allows us to employ Bayes factor testing in the identification of the most parsimonious description of the diffusion process [8]. We used a modified Association Index (AI) to assess the degree of spatial structure in the phylogeographic data [8], [15]. This reports the posterior distribution of association values relative to those obtained by randomizing the tip locations. In addition, we summarize the number of transitions to each sampling location in the posterior tree distribution based on the location realizations at the nodes. The latter provide a conservative estimate of the number of independent introductions in each location.

To quantify the dissemination process, we estimated the rate of rabies spread among the sampled isolates using ‘Markov jump’ counts [19] of location state transitions for all possible states along the phylogeny. Markov jump counts measure the expected number of transitions along each branch conditional on the observed data. By multiplying the expected number of transitions between each pair of locations by the geographical distance between these two locations, we arrive at the expected distance travelled within the time elapsed on each branch. This approach, implemented in BEAGLE [41] a library that can be used in conjunction with BEAST), integrates over all uncertainty in the evolutionary tree and offers a degree of robustness to model misspecification [42].

To test different scenarios of phylogeographic diffusion, we fix the CTMC relative rate parameters to the normalized pairwise location measures that represent different diffusion predictors and perform Bayesian model selection using marginal likelihood approximations [43]. We consider; (i) geographical distances, specifically great-circle distances that represent the shortest path on the surface of the Earth between two points, (ii) human population size, obtained from http://en.wikipedia.org/ and http://www.mongabay.com (Table S3 in Supporting Information S1, rates between each pair of locations were fixed to the normalized products of the population sizes), (iii) road distances, (iv) a gravity model, (v) spatial accessibility, and (vi) ‘population conductivity’ measures. The accessibility estimates are derived from a range of spatial data sets, road type and network data [44], satellite derived and cover information, settlement database locations and sizes, and satellite derived topography. They are combined to create a ‘friction surface’ where each 1×1 km square represents the difficulty (or travel time) in crossing it. These estimates provide a representation of the difficulty in travel between all the locations. Using a circuit theory approach, an origin-destination distance matrix was estimated from this accessibility landscape [17]. A simple gravity model was constructed by fixing the rates to the normalized product of the population sizes divided by the great circle distance between the locations involved. As an alternative, population sizes were also mapped in a landscape (Figure S2 in Supporting Information S1) [18] and again translated to an origin-destination distance matrix using circuit theory. To assess model fit, marginal likelihood approximations are obtained using an importance sampling estimator [43], [45], which employs a mixture of model prior and posterior samples [46].

### Spatial simulation

To contrast the spatial distribution of our rabies samples in Algeria and Morocco with the patterns of spread we would expect from local transmission dynamics in African dogs as the sole maintenance population, we performed a simulation analysis that integrates phylodynamic and epidemiological parameters. Specifically, we consider a spatial process based on epidemiological parameters obtained from a detailed analysis of rabies transmission biology in African dogs [20], and simulate virus movement accordingly over all evolutionary histories in the posterior distribution resulting from our phylodynamic inference. The latter characterizes the successful ancestral transmission history of the viruses we sampled and provides a time-scale for the spatial process we would like to simulate.

For each tree in the posterior distribution, we consider the ancestral virus at the root to start spreading from the mid-point of our available samples (average of longitudes and latitudes). We recursively visit all branches from root to tip, each time simulating a number of successive infections, which jointly encompass the entire time length for each branch. Each time interval *t* (in days) between successive infections follows:
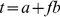
(1)where *a* is a random incubation time, *b* is the random period of infectiousness, and *f* is a random fraction drawn from a uniform[0,1] distribution. Following the results of the comprehensive study by Hampson et al. [21], we consider
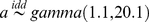
(2)and
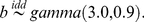
(3)Each new infection is moved a random distance *d* (in m) away from its source case; the distribution for *d* follows a previously described spatial infection kernel [21]:
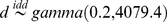
(4)In the spatial simulation process, we assume that each new distance takes a random direction in continuous space, but we also explore subsequent infections consistently taking the same direction. To achieve a realistic distribution in the relevant geographic area, we prohibit new infections to invade water areas. The tree heights used for simulation for Algeria and Morocco were 33 (23–46) years and 28 (18–39) years respectively (as estimated from the country-specific data), whereas the tree lengths encompassed 623 (490–789) years and 532 (367–706) years respectively.

This simulation procedure yields location realizations in continuous space for all tips and all trees in the posterior distribution. We summarize this spatial distribution using two-dimensional contours. Because the spatial simulation for each tree in the posterior distribution may cover a different area, it is important to note that the contour representing the process over all trees depicts the maximum area that can be covered for the set of epidemiological parameters we consider.

## Supporting Information

Supporting Information S1 Tables S1, S2, S3 and Figures S1 and S2
(4.34 MB DOC)
