# Correction: Phylodynamics and Human-Mediated Dispersal of a Zoonotic Virus

**DOI:** 10.1371/annotation/66c3b26f-7b78-4412-96d0-c960b8d74b50

**Published:** 2011-01-26

**Authors:** Chiraz Talbi, Philippe Lemey, Marc A. Suchard, Elbia Abdelatif, Mehdi Elharrak, Jalal Nourlil, Abdellah Faouzi, Juan E. Echevarría, Sonia Vazquez Morón, Andrew Rambaut, Nicholas Campiz, Andrew J. Tatem, Edward C. Holmes, Hervé Bourhy

The sixth author's name was written incorrectly. The correct name is: Jalal Nourlil. In the author contributions, "Performed the experiments" should read: CT PL MAS EA ME JN AF JEE SVM AR NC AJT ECH HB; "Analyzed the data" should read: CT PL MAS EA ME JN AF JEE SVM AR NC AJT ECH HB; "Contributed reagents/materials/analysis tools" should read: CT PL MAS EA ME JN AF JEE SVM AR NC AJT ECH HB; and "Wrote the paper" should read: CT PL MAS EA ME JN AF JEE SVM AR NC AJT ECH HB.

