# Supplementary material for: Phylodynamics and Human-Mediated Dispersal of a Zoonotic Virus
Source: PLoS Pathog. 2010 Oct 28;6(10):e1001166. doi: 10.1371/journal.ppat.1001166 (PMC2965766; doi:10.1371/journal.ppat.1001166)

**Supplementary tables and figures**

**Table S1**. Relevant epidemiological information for all RABV isolates analysed in this study

| **Countries** | **Isolates** | **Cities** | **Species** | **Dates** | **Latitude** | **Longitude** |
| --- | --- | --- | --- | --- | --- | --- |
| Morocco | 08-342 | Importation Morocco | Dog | 9/11/04 | 34°32'8.88"N | 4°38'24.36"W |
| Morocco | 9107 | Ouezzane | Human | 30/06/86 | 34°48'28.44"N | 5°34'23.88"W |
| Morocco | 08-337 | Importation Morocco | Dog | 18/02/04 | 30° 9'0.00"N | 8°58'0.00"W |
| Morocco | 9016 | Importation Morocco | Human | 2/05/86 |  |  |
| Morocco | 9109 | Marrakech | Human | 30/06/86 | 31°38'21.01"N | 8° 0'17.17"W |
| Morocco | 9106 | Marrakech | Human | 30/06/86 | 31°38'21.01"N | 8° 0'17.17"W |
| Morocco | 9108 | Marrakech | Human | 30/06/86 | 31°38'21.01"N | 8° 0'17.17"W |
| Morocco | 8678 | NO DATA | Human | 1986 |  |  |
| Morocco | 09-001 | Rabat | Human | 25/05/97 | 33°59'25.80"N | 6°52'13.44"W |
| Morocco | 09-002 | Tanger | Human | 15/07/97 | 35°46'50.52"N | 5°48'41.04"W |
| Morocco | 09-003 | Casa | Human | 18/12/99 | 33°35'20.04"N | 7°36'32.04"W |
| Morocco | 09-004 | Tanger | Human | 9/05/00 | 35°46'50.52"N | 5°48'41.04"W |
| Morocco | 09-005 | Oujda | Human | 18/06/00 | 34°41'39.48"N | 1°55'5.88"W |
| Morocco | 09-006 | Settat | Human | 10/10/00 | 33°0'22.32"N | 7°37'9.48"W |
| Morocco | 08-001 | Chichaoua | Dog | 8/01/03 | 31°32'47.40"N | 8°45'45.36"W |
| Morocco | 08-002 | Azilal | caprine | 18/01/03 | 31°58'0.84"N | 6°33'58.32"W |
| Morocco | 08-003 | Marrakech | Dog | 25/01/03 | 31°38'21.01"N | 8° 0'17.17"W |
| Morocco | 08-004 | Ouarzazate | Equine | 28/01/03 | 30°55'8.00"N | 6°54'13.75"W |
| Morocco | 08-005 | Marrakech | Equine | 15/02/03 | 31°38'21.01"N | 8° 0'17.17"W |
| Morocco | 08-006 | Ouarzazate | Dog | 21/02/03 | 30°55'8.00"N | 6°54'13.75"W |
| Morocco | 08-007 | Ouarzazate | Dog | 21/02/03 | 30°55'8.00"N | 6°54'13.75"W |
| Morocco | 08-008 | Ouarzazate | Equine | 21/02/03 | 30°55'8.00"N | 6°54'13.75"W |
| Morocco | 08-009 | Chichaoua | Bovine | 22/02/03 | 31°32'47.40"N | 8°45'45.36"W |
| Morocco | 08-010 | Marrakech | Bovine | 11/03/03 | 31°38'21.01"N | 8° 0'17.17"W |
| Morocco | 08-011 | Marrakech | Dog | 28/03/03 | 31°38'21.01"N | 8° 0'17.17"W |
| Morocco | 08-012 | Ouarzazate | Equine | 2/04/03 | 30°55'8.00"N | 6°54'13.75"W |
| Morocco | 08-013 | Marrakech | Dog | 8/04/03 | 31°38'21.01"N | 8° 0'17.17"W |
| Morocco | 08-014 | Marrakech | Donkey | 13/05/03 | 31°38'21.01"N | 8° 0'17.17"W |
| Morocco | 08-015 | Marrakech | Dog | 31/05/03 | 31°38'21.01"N | 8° 0'17.17"W |
| Morocco | 08-016 | Marrakech | Dog | 5/06/03 | 31°38'21.01"N | 8° 0'17.17"W |
| Morocco | 08-017 | Marrakech | Dog | 3/07/03 | 31°38'21.01"N | 8° 0'17.17"W |
| Morocco | 08-018 | Beni mellal | Donkey | 23/07/03 | 32°20'8.81"N | 6°21'21.56"W |
| Morocco | 08-019 | Marrakech | Dog | 13/09/03 | 31°38'21.01"N | 8° 0'17.17"W |
| Morocco | 08-020 | Marrakech | Donkey | 5/10/03 | 31°38'21.01"N | 8° 0'17.17"W |
| Morocco | 08-021 | Marrakech | Dog | 30/10/03 | 31°38'21.01"N | 8° 0'17.17"W |
| Morocco | 08-022 | Azilal | Bovine | 1/11/03 | 31°58'0.84"N | 6°33'58.32"W |
| Morocco | 08-023 | Marrakech | Dog | 6/11/03 | 31°38'21.01"N | 8° 0'17.17"W |
| Morocco | 08-024 | Ouarzazate | Sheep | 19/11/03 | 30°55'8.00"N | 6°54'13.75"W |
| Morocco | 08-025 | Tanger | Bovine | 22/02/00 | 35°46'50.52"N | 5°48'41.04"W |
| Morocco | 08-031 | Sidi kacem | Dog | 14/10/00 | 33°16'60.00"N | 7°52'0.00"W |
| Morocco | 08-033 | Kenitra | Dog | 10/11/00 | 34°16'22.44"N | 6°34'33.60"W |
| Morocco | 08-034 | Tanger | Dog | 21/11/00 | 35°46'50.52"N | 5°48'41.04"W |
| Morocco | 08-035 | Kenitra | Bovine | 22/11/00 | 34°16'22.44"N | 6°34'33.60"W |
| Morocco | 08-038 | Sidi kacem | Bovine | 7/04/01 | 33°16'60.00"N | 7°52'0.00"W |
| Morocco | 08-039 | Kenitra | Bovine | 7/04/01 | 34°16'22.44"N | 6°34'33.60"W |
| Morocco | 08-040 | Casa | Equine | 1/10/02 | 33°35'20.04"N | 7°36'32.04"W |
| Morocco | 08-041 | Benslimane | Donkey | 23/11/02 | 33°36'35.28"N | 7° 7'16.32"W |
| Morocco | 08-043 | Casa | Dog | 21/01/03 | 33°35'20.04"N | 7°36'32.04"W |
| Morocco | 08-045 | Benslimane | Dog | 19/02/03 | 33°36'35.28"N | 7° 7'16.32"W |
| Morocco | 08-046 | Benslimane | Bovine | 19/02/03 | 33°36'35.28"N | 7° 7'16.32"W |
| Morocco | 08-047 | Casa | Dog | 25/02/03 | 33°35'20.04"N | 7°36'32.04"W |
| Morocco | 08-048 | Settat | Dog | 25/03/03 | 33°0'22.32"N | 7°37'9.48"W |
| Morocco | 08-049 | Benslimane | Bovine | 4/04/03 | 33°36'35.28"N | 7° 7'16.32"W |
| Morocco | 08-050 | Settat | Dog | 9/04/03 | 33°0'22.32"N | 7°37'9.48"W |
| Morocco | 08-051 | Benslimane | Dog | 10/04/03 | 33°36'35.28"N | 7° 7'16.32"W |
| Morocco | 08-052 | Khouribga | Dog | 24/04/03 | 32°52'51.60"N | 6°54'39.96"W |
| Morocco | 08-053 | Benslimane | Bovine | 1/05/03 | 33°36'35.28"N | 7° 7'16.32"W |
| Morocco | 08-054 | Casa | Dog | 6/05/03 | 33°35'20.04"N | 7°36'32.04"W |
| Morocco | 08-055 | Casa | Dog | 10/05/03 | 33°35'20.04"N | 7°36'32.04"W |
| Morocco | 08-056 | Settat | Dog | 27/07/01 | 33°0'22.32"N | 7°37'9.48"W |
| Morocco | 08-057 | Settat | Sheep | 20/06/03 | 33°0'22.32"N | 7°37'9.48"W |
| Morocco | 08-058 | Casa | Dog | 11/07/03 | 33°35'20.04"N | 7°36'32.04"W |
| Morocco | 08-059 | Nador | Dog | 4/01/03 | 33°37'0.00"N | 3°44'0.00"W |
| Morocco | 08-060 | Berkane | Bovine | 4/01/03 | 34°55'28.56"N | 2°20'2.40"W |
| Morocco | 08-061 | Berkane | Donkey | 1/02/03 | 34°55'28.56"N | 2°20'2.40"W |
| Morocco | 08-062 | Nador | Dog | 8/02/03 | 33°37'0.00"N | 3°44'0.00"W |
| Morocco | 08-063 | Oujda | Dog | 21/02/03 | 34°41'39.48"N | 1°55'5.88"W |
| Morocco | 08-064 | Berkane | Dog | 25/02/03 | 34°55'28.56"N | 2°20'2.40"W |
| Morocco | 08-065 | Al hoceima | Dog | 12/03/03 | 35°14'57.48"N | 3°56'13.60"W |
| Morocco | 08-066 | Jerrada | Dog | 13/03/03 | 34°18'36.00"N | 2° 9'38.88"W |
| Morocco | 08-067 | Taourirt | Donkey | 22/04/03 | 32°41'57.01"N | 9° 4'28.79"W |
| Morocco | 08-068 | Berkane | Dog | 20/05/03 | 34°55'28.56"N | 2°20'2.40"W |
| Morocco | 08-069 | Berkane | Donkey | 30/05/03 | 34°55'28.56"N | 2°20'2.40"W |
| Morocco | 08-070 | Oujda | Dog | 17/06/03 | 34°41'39.48"N | 1°55'5.88"W |
| Morocco | 08-071 | Berkane | Dog | 27/06/03 | 34°55'28.56"N | 2°20'2.40"W |
| Morocco | 08-072 | Berkane | Bovine | 9/09/03 | 34°55'28.56"N | 2°20'2.40"W |
| Morocco | 08-073 | Taourirt | Dog | 2/08/03 | 32°41'57.01"N | 9° 4'28.79"W |
| Morocco | 08-074 | Berkane | Bovine | 8/08/03 | 34°55'28.56"N | 2°20'2.40"W |
| Morocco | 08-075 | Taourirt | Donkey | 12/08/03 | 32°41'57.01"N | 9° 4'28.79"W |
| Morocco | 08-076 | Taourirt | Equine | 12/08/03 | 32°41'57.01"N | 9° 4'28.79"W |
| Morocco | 08-077 | Oujda | Sheep | 26/08/03 | 34°41'39.48"N | 1°55'5.88"W |
| Morocco | 08-078 | Oujda | caprine | 14/11/03 | 34°41'39.48"N | 1°55'5.88"W |
| Morocco | 08-079 | Rabat | Equine | 2/01/03 | 33°59'25.80"N | 6°52'13.44"W |
| Morocco | 08-080 | Rabat | Dog | 3/05/03 | 33°59'25.80"N | 6°52'13.44"W |
| Morocco | 08-081 | Rabat | Dog | 6/05/03 | 33°59'25.80"N | 6°52'13.44"W |
| Morocco | 08-082 | Rabat | Dog | 27/11/02 | 33°59'25.80"N | 6°52'13.44"W |
| Morocco | 08-083 | Rabat | Dog | 20/12/02 | 33°59'25.80"N | 6°52'13.44"W |
| Morocco | 08-084 | Rabat | Dog | 26/07/03 | 33°59'25.80"N | 6°52'13.44"W |
| Morocco | 08-085 | Rabat | Dog | 5/07/03 | 33°59'25.80"N | 6°52'13.44"W |
| Morocco | 08-086 | Sidi kacem | Dog | 21/01/03 | 33°16'60.00"N | 7°52'0.00"W |
| Morocco | 08-087 | Sidi kacem | Dog | 30/01/03 | 33°16'60.00"N | 7°52'0.00"W |
| Morocco | 08-089 | Errachidia | Dog | 2/04/03 | 31°56'41.06"N | 4°24'2.16"W |
| Morocco | 08-090 | El hajeb | Bovine | 8/04/03 | 33°41'53.16"N | 5°21'53.23"W |
| Morocco | 08-091 | Errachidia | Dog | 9/04/03 | 31°56'41.06"N | 4°24'2.16"W |
| Morocco | 08-092 | Figuig | Mule | 9/04/03 | 32°6'3.60"N | 1°13'17.04"W |
| Morocco | 08-093 | Taza | Bovine | 9/04/03 | 34°13'58.79"N | 3°56'57.03"W |
| Morocco | 08-094 | Khenifra | Bovine | 11/04/03 | 32°56'18.96"N | 5°40'5.52"W |
| Morocco | 08-095 | Sidi kacem | Dog | 12/04/03 | 33°16'60.00"N | 7°52'0.00"W |
| Morocco | 08-096 | Sidi kacem | Bovine | 23/04/03 | 33°16'60.00"N | 7°52'0.00"W |
| Morocco | 08-097 | Taza | Dog | 23/04/03 | 34°13'58.79"N | 3°56'57.03"W |
| Morocco | 08-098 | Sidi kacem | Bovine | 6/05/03 | 33°16'60.00"N | 7°52'0.00"W |
| Morocco | 08-099 | Ifrane | Bovine | 8/05/03 | 34°2'60.00"N | 3°46'0.00"W |
| Morocco | 08-100 | Errachidia | Dog | 20/05/03 | 31°56'41.06"N | 4°24'2.16"W |
| Morocco | 08-101 | Sidi kacem | Bovine | 21/06/03 | 33°16'60.00"N | 7°52'0.00"W |
| Morocco | 08-102 | Errachidia | Dog | 26/06/03 | 31°56'41.06"N | 4°24'2.16"W |
| Morocco | 08-103 | Fès | Bovine | 5/07/03 | 34°2'12.70"N | 5° 0'10.01"W |
| Morocco | 08-104 | Figuig | Sheep | 9/07/03 | 32°6'3.60"N | 1°13'17.04"W |
| Morocco | 08-105 | El hajeb | Dog | 11/07/03 | 33°41'53.16"N | 5°21'53.23"W |
| Morocco | 08-106 | Taounate | Bovine | 22/07/03 | 34°32'8.88"N | 4°38'24.36"W |
| Morocco | 08-110 | Khenifra | Mule | 2/10/03 | 32°56'18.96"N | 5°40'5.52"W |
| Morocco | 08-111 | Agadir | Dog | 20/02/03 | 30° 9'0.00"N | 8°58'0.00"W |
| Morocco | 08-112 | Agadir | Bovine | 1/04/03 | 30° 9'0.00"N | 8°58'0.00"W |
| Morocco | 08-113 | Agadir | Cat | 11/07/03 | 30° 9'0.00"N | 8°58'0.00"W |
| Morocco | 08-114 | Agadir | Dog | 21/05/02 | 30° 9'0.00"N | 8°58'0.00"W |
| Morocco | 08-116 | Agadir | Dog | 14/08/02 | 30° 9'0.00"N | 8°58'0.00"W |
| Morocco | 08-117 | Agadir | Dog | 27/03/03 | 30° 9'0.00"N | 8°58'0.00"W |
| Morocco | 08-118 | Agadir | Dog | 29/06/02 | 30° 9'0.00"N | 8°58'0.00"W |
| Morocco | 08-119 | Agadir | Dog | 20/12/00 | 30° 9'0.00"N | 8°58'0.00"W |
| Morocco | 08-297 | Sidi kacem | Bovine | 15/03/01 | 33°16'60.00"N | 7°52'0.00"W |
| Morocco | 08-298 | Tanger | Bovine | 26/04/00 | 35°46'50.52"N | 5°48'41.04"W |
| Morocco | 08-299 | Tanger | Bovine | 13/07/00 | 35°46'50.52"N | 5°48'41.04"W |
| Morocco | 08-302 | Tanger | Bovine | 6/01/01 | 35°46'50.52"N | 5°48'41.04"W |
| Morocco | 08-303 | Tanger | Bovine | 27/07/00 | 35°46'50.52"N | 5°48'41.04"W |
| Morocco | 08-304 | Tanger | Bovine | 2/08/00 | 35°46'50.52"N | 5°48'41.04"W |
| Morocco | 08-305 | Tanger | Dog | 11/08/00 | 35°46'50.52"N | 5°48'41.04"W |
| Morocco | 08-306 | Tanger | Bovine | 26/02/03 | 35°46'50.52"N | 5°48'41.04"W |
| Morocco | 08-307 | Tanger | Bovine | 13/07/00 | 35°46'50.52"N | 5°48'41.04"W |
| Morocco | 08-311 | Benslimane | Bovine | 30/04/03 | 33°36'35.28"N | 7° 7'16.32"W |
| Morocco | 08-312 | Benslimane | Dog | 30/04/03 | 33°36'35.28"N | 7° 7'16.32"W |
| Morocco | 08-314 | Casa | Dog | 29/02/04 | 33°35'20.04"N | 7°36'32.04"W |
| Morocco | 08-316 | Casa | Bovine | 29/02/04 | 33°35'20.04"N | 7°36'32.04"W |
| Morocco | 08-317 | Benslimane | Dog | 31/03/04 | 33°36'35.28"N | 7° 7'16.32"W |
| Morocco | 08-318 | Oujda | Dog | 21/02/04 | 34°41'39.48"N | 1°55'5.88"W |
| Morocco | 08-319 | Al hoceima | Dog | 15/04/04 | 35°14'57.48"N | 3°56'13.60"W |
| Morocco | 08-320 | Berkane | Dog | 28/04/04 | 34°55'28.56"N | 2°20'2.40"W |
| Morocco | 08-322 | Marrakech | Dog | 31/01/03 | 31°38'21.01"N | 8° 0'17.17"W |
| Morocco | 08-323 | Ouarzazate | Dog | 31/01/03 | 30°55'8.00"N | 6°54'13.75"W |
| Morocco | 08-324 | Marrakech | Dog | 31/01/03 | 31°38'21.01"N | 8° 0'17.17"W |
| Morocco | 08-325 | Chichaoua | Bovine | 1/03/07 | 31°32'47.40"N | 8°45'45.36"W |
| Morocco | 08-326 | Marrakech | Dog | 31/05/03 | 31°38'21.01"N | 8° 0'17.17"W |
| Morocco | 08-327 | Marrakech | Dog | 31/03/03 | 31°38'21.01"N | 8° 0'17.17"W |
| Morocco | 08-328 | Marrakech | Dog | 30/04/03 | 31°38'21.01"N | 8° 0'17.17"W |
| Morocco | 08-329 | Beni mellal | Donkey | 30/04/03 | 32°20'8.81"N | 6°21'21.56"W |
| Morocco | 08-330 | Marrakech | Dog | 30/09/03 | 31°38'21.01"N | 8° 0'17.17"W |
| Morocco | 08-333 | Beni mellal | Bovine | 31/03/03 | 32°20'8.81"N | 6°21'21.56"W |
| Morocco | 08-334 | Marrakech | Donkey | 31/05/03 | 31°38'21.01"N | 8° 0'17.17"W |
| Morocco | 08-335 | Marrakech | Dog | 31/05/03 | 31°38'21.01"N | 8° 0'17.17"W |
| Algeria | 9137ALG | NO DATA | Dog | 1991 |  |  |
| Algeria | 9618ALG | NO DATA | Human | 1996 |  |  |
| Algeria | 9619ALG | NO DATA | Human | 1996 |  |  |
| Algeria | 08-121 | Alger | Dog | 9/01/03 | 36°46'31.80"N | 3° 3'34.92"E |
| Algeria | 08-122 | Blida | Dog | 9/01/03 | 36°28'22.44"N | 2°49'30.36"E |
| Algeria | 08-123 | blida | Jackal | 12/01/03 | 36°28'22.44"N | 2°49'30.36"E |
| Algeria | 08-124 | Ain Defla | Dog | 9/02/03 | 36°4'2.36"N | 4°33'19.09"E |
| Algeria | 08-125 | Bejaia | Dog | 12/02/03 | 36°32'34.05"N | 4°58'26.17"E |
| Algeria | 08-127 | Msila | Dog | 3/03/03 | 35°7'8.31"N | 4°25'44.65"E |
| Algeria | 08-128 | Bouira | Dog | 15/03/03 | 36°12'30.32"N | 3°55'30.18"E |
| Algeria | 08-129 | Msila | Dog | 22/03/03 | 35°7'8.31"N | 4°25'44.65"E |
| Algeria | 08-130 | Tipaza | Dog | 1/04/03 | 36°34'24.91"N | 2°25'15.83"E |
| Algeria | 08-131 | Bejaia | Dog | 16/04/03 | 36°32'34.05"N | 4°58'26.17"E |
| Algeria | 08-132 | Biskra | Dog | 27/04/03 | 34°51'20.16"N | 5°43'41.16"E |
| Algeria | 08-133 | Alger | Dog | 27/04/03 | 36°46'31.80"N | 3° 3'34.92"E |
| Algeria | 08-134 | Bejaia | Dog | 27/04/03 | 36°32'34.05"N | 4°58'26.17"E |
| Algeria | 08-135 | Blida | Sheep | 11/05/03 | 36°28'22.44"N | 2°49'30.36"E |
| Algeria | 08-136 | Djelfa | Donkey | 22/05/03 | 34°40'32.52"N | 3°15'7.20"E |
| Algeria | 08-137 | Bouira | Dog | 28/05/03 | 36°12'30.32"N | 3°55'30.18"E |
| Algeria | 08-139 | Tissemsilt | Dog | 1/06/03 | 35°45'9.94"N | 1°52'2.06"E |
| Algeria | 08-140 | Bouira | Dog | 3/06/03 | 36°12'30.32"N | 3°55'30.18"E |
| Algeria | 08-141 | Chlef | Cow | 8/06/03 | 36°9'54.36"N | 1°19'37.20"E |
| Algeria | 08-142 | Tipaza | Dog | 10/06/03 | 36°34'24.91"N | 2°25'15.83"E |
| Algeria | 08-143 | Boumerdes | Dog | 10/06/03 | 36°43'35.34"N | 3°42'35.48"E |
| Algeria | 08-144 | Relizane | Donkey | 16/06/03 | 35°44'37.68"N | 0°33'29.88"E |
| Algeria | 08-145 | chlef | Cow | 18/06/03 | 36°9'54.36"N | 1°19'37.20"E |
| Algeria | 08-146 | Médéa | Cat | 22/06/03 | 36°15'46.80"N | 2°45'29.52"E |
| Algeria | 08-147 | Borj Bou arreridj | Goat | 17/04/00 | 35°59'.35"N | 4°58'.1"W |
| Algeria | 08-149 | Médéa | Dog | 13/05/00 | 36°15'46.80"N | 2°45'29.52"E |
| Algeria | 08-150 | Ouargla | Dog | 29/11/01 | 31°56'44.52"N | 5°19'31.44"E |
| Algeria | 08-151 | Tizi-Ouzou | Dog | 26/06/03 | 36°33'.21"N | 4°5'.22"E |
| Algeria | 08-152 | Msila | Dog | 3/07/03 | 35°7'8.31"N | 4°25'44.65"E |
| Algeria | 08-153 | Constantine | Dog | 8/07/03 | 36°21'46.44"N | 6°36'31.32"E |
| Algeria | 08-154 | Blida | Jackal | 19/05/00 | 36°15'46.80"N | 2°45'29.52"E |
| Algeria | 08-155 | sétif | Dog | 9/07/03 | 36°11'18.96"N | 5°24'25.92"E |
| Algeria | 08-156 | Tissemsilt | Dog | 13/02/01 | 35°45'9.94"N | 1°52'2.06"E |
| Algeria | 08-157 | Alger | Dog | 17/07/03 | 36°46'31.80"N | 3° 3'34.92"E |
| Algeria | 08-159 | Boumerdes | Sheep | 23/07/03 | 36°43'35.34"N | 3°42'35.48"E |
| Algeria | 08-161 | Tipaza | Dog | 30/07/03 | 36°34'24.91"N | 2°25'15.83"E |
| Algeria | 08-162 | Borj Bou arreridj | Bovine | 5/08/03 | 35°59'.35"N | 4°58'.1"W |
| Algeria | 08-165 | Alger | Goat | 15/08/03 | 36°46'31.80"N | 3° 3'34.92"E |
| Algeria | 08-166 | Blida | Dog | 17/08/03 | 36°28'22.44"N | 2°49'30.36"E |
| Algeria | 08-167 | Médéa | Dog | 10/09/03 | 36°15'46.80"N | 2°45'29.52"E |
| Algeria | 08-170 | Chlef | Cat | 29/09/03 | 36°9'54.36"N | 1°19'37.20"E |
| Algeria | 08-172 | Bouira | Dog | 3/11/03 | 36°12'30.32"N | 3°55'30.18"E |
| Algeria | 08-173 | Chlef | Dog | 4/11/03 | 36°9'54.36"N | 1°19'37.20"E |
| Algeria | 08-174 | Djelfa | Dog | 7/12/03 | 34°40'32.52"N | 3°15'7.20"E |
| Algeria | 08-175 | Alger | Cat | 10/12/03 | 36°46'31.80"N | 3° 3'34.92"E |
| Algeria | 08-176 | Msila | Donkey | 21/12/03 | 35°7'8.31"N | 4°25'44.65"E |
| Algeria | 08-177 | Alger | Dog | 24/12/03 | 36°46'31.80"N | 3° 3'34.92"E |
| Algeria | 08-178 | Alger | Dog | 24/12/03 | 36°46'31.80"N | 3° 3'34.92"E |
| Algeria | 08-180 | Alger | Dog | 7/01/04 | 36°46'31.80"N | 3° 3'34.92"E |
| Algeria | 08-181 | Alger | Donkey | 8/01/04 | 36°46'31.80"N | 3° 3'34.92"E |
| Algeria | 08-183 | Médéa | Dog | 21/01/04 | 36°15'46.80"N | 2°45'29.52"E |
| Algeria | 08-185 | Blida | Sheep | 25/01/04 | 36°28'22.44"N | 2°49'30.36"E |
| Algeria | 08-187 | Boumerdes | Dog | 2/02/04 | 36°43'35.34"N | 3°42'35.48"E |
| Algeria | 08-188 | Relizane | Dog | 5/02/04 | 35°44'37.68"N | 0°33'29.88"E |
| Algeria | 08-189 | Alger | Dog | 5/02/04 | 36°46'31.80"N | 3° 3'34.92"E |
| Algeria | 08-190 | Bejaia | Sheep | 11/02/04 | 36°32'34.05"N | 4°58'26.17"E |
| Algeria | 08-191 | Ain Defla | Dog | 12/02/04 | 36°4'2.36"N | 4°33'19.09"E |
| Algeria | 08-192 | Msila | Dog | 11/02/04 | 35°7'8.31"N | 4°25'44.65"E |
| Algeria | 08-193 | Alger | Dog | 20/02/04 | 36°46'31.80"N | 3° 3'34.92"E |
| Algeria | 08-194 | Tissemsilt | Dog | 24/02/04 | 35°45'9.94"N | 1°52'2.06"E |
| Algeria | 08-195 | Alger | Dog | 25/02/04 | 36°46'31.80"N | 3° 3'34.92"E |
| Algeria | 08-196 | Blida | Cat | 28/02/04 | 36°28'22.44"N | 2°49'30.36"E |
| Algeria | 08-197 | Boumerdes | Cat | 2/03/04 | 36°43'35.34"N | 3°42'35.48"E |
| Algeria | 08-198 | Alger | Dog | 4/03/04 | 36°46'31.80"N | 3° 3'34.92"E |
| Algeria | 08-199 | Ain Defla | caprine | 12/03/04 | 36°4'2.36"N | 4°33'19.09"E |
| Algeria | 08-200 | sétif | Human | 13/03/04 | 36°11'18.96"N | 5°24'25.92"E |
| Algeria | 08-201 | Mascara | Dog | 12/03/04 | 35°40'57.90"N | 0°8'37.86"W |
| Algeria | 08-202 | Alger | Dog | 16/03/04 | 36°46'31.80"N | 3° 3'34.92"E |
| Algeria | 08-203 | Tipaza | Dog | 17/03/04 | 36°34'24.91"N | 2°25'15.83"E |
| Algeria | 08-204 | Alger | Dog | 28/03/04 | 36°46'31.80"N | 3° 3'34.92"E |
| Algeria | 08-205 | Tipaza | Cow | 2/04/04 | 36°34'24.91"N | 2°25'15.83"E |
| Algeria | 08-206 | sétif | Human | 3/04/04 | 36°11'18.96"N | 5°24'25.92"E |
| Algeria | 08-207 | Alger | Dog | 9/04/04 | 36°46'31.80"N | 3° 3'34.92"E |
| Algeria | 08-208 | Tipaza | Dog | 11/04/04 | 36°34'24.91"N | 2°25'15.83"E |
| Algeria | 08-209 | Alger | Cat | 22/12/97 | 36°46'31.80"N | 3° 3'34.92"E |
| Algeria | 08-210 | Alger | Dog | 30/11/97 | 36°46'31.80"N | 3° 3'34.92"E |
| Algeria | 08-211 | Blida | Dog | 19/12/97 | 36°28'22.44"N | 2°49'30.36"E |
| Algeria | 08-212 | Médéa | Cow | 15/07/97 | 36°15'46.80"N | 2°45'29.52"E |
| Algeria | 08-214 | Bejaia | Dog | 24/11/97 | 36°32'34.05"N | 4°58'26.17"E |
| Algeria | 08-215 | Blida | Cow | 22/11/97 | 36°28'22.44"N | 2°49'30.36"E |
| Algeria | 08-216 | Tizi-Ouzou | Dog | 12/05/97 | 36°33'.21"N | 4°5'.22"E |
| Algeria | 08-217 | Tipaza | Dog | 17/04/97 | 36°34'24.91"N | 2°25'15.83"E |
| Algeria | 08-218 | Boumerdes | Cat | 5/11/97 | 36°43'35.34"N | 3°42'35.48"E |
| Algeria | 08-219 | Blida | Cow | 28/10/97 | 36°28'22.44"N | 2°49'30.36"E |
| Algeria | 08-220 | Tizi-Ouzou | Cow | 22/05/97 | 36°33'.21"N | 4°5'.22"E |
| Algeria | 08-221 | Borj Bou arreridj | Dog | 1/03/98 | 35°59'.35"N | 4°58'.1"W |
| Algeria | 08-222 | Boumerdes | Dog | 21/05/98 | 36°43'35.34"N | 3°42'35.48"E |
| Algeria | 08-223 | Médéa | Dog | 12/06/98 | 36°15'46.80"N | 2°45'29.52"E |
| Algeria | 08-224 | Alger | Dog | 22/09/98 | 36°46'31.80"N | 3° 3'34.92"E |
| Algeria | 08-225 | Boumerdes | Dog | 28/09/98 | 36°43'35.34"N | 3°42'35.48"E |
| Algeria | 08-226 | Tipaza | Dog | 21/04/98 | 36°34'24.91"N | 2°25'15.83"E |
| Algeria | 08-227 | Blida | Dog | 31/05/98 | 36°28'22.44"N | 2°49'30.36"E |
| Algeria | 08-228 | Msila | Sheep | 14/05/98 | 35°7'8.31"N | 4°25'44.65"E |
| Algeria | 08-229 | Boumerdes | Dog | 5/04/98 | 36°28'22.44"N | 2°49'30.36"E |
| Algeria | 08-230 | Batna | Cow | 3/02/98 | 35°33'.19"N | 6°10'.42"E |
| Algeria | 08-231 | Tipaza | Dog | 26/02/98 | 36°34'24.91"N | 2°25'15.83"E |
| Algeria | 08-233 | Médéa | Jackal | 25/02/99 | 36°15'46.80"N | 2°45'29.52"E |
| Algeria | 08-234 | Blida | Goat | 13/07/99 | 36°28'22.44"N | 2°49'30.36"E |
| Algeria | 08-235 | Alger | Cat | 24/05/99 | 36°46'31.80"N | 3° 3'34.92"E |
| Algeria | 08-236 | Tipaza | Dog | 15/04/99 | 36°34'24.91"N | 2°25'15.83"E |
| Algeria | 08-237 | Bouira | Dog | 1/03/99 | 36°12'30.32"N | 3°55'30.18"E |
| Algeria | 08-239 | Médéa | Sheep | 25/03/99 | 36°15'46.80"N | 2°45'29.52"E |
| Algeria | 08-240 | Boumerdes | Sheep | 29/03/99 | 36°43'35.34"N | 3°42'35.48"E |
| Algeria | 08-241 | Alger | Cat | 16/08/99 | 36°46'31.80"N | 3° 3'34.92"E |
| Algeria | 08-242 | Alger | Dog | 9/05/99 | 36°46'31.80"N | 3° 3'34.92"E |
| Algeria | 08-243 | Relizane | Calf | 25/03/99 | 35°44'37.68"N | 0°33'29.88"E |
| Algeria | 08-245 | Blida | Calf | 1/04/99 | 36°28'22.44"N | 2°49'30.36"E |
| Algeria | 08-246 | Chlef | Dog | 11/05/99 | 36°9'54.36"N | 1°19'37.20"E |
| Algeria | 08-247 | Blida | Dog | 18/02/99 | 36°28'22.44"N | 2°49'30.36"E |
| Algeria | 08-248 | Alger | Dog | 30/11/00 | 36°46'31.80"N | 3° 3'34.92"E |
| Algeria | 08-249 | Alger | Dog | 14/05/00 | 36°46'31.80"N | 3° 3'34.92"E |
| Algeria | 08-250 | Tipaza | Bovine | 17/08/00 | 36°34'24.91"N | 2°25'15.83"E |
| Algeria | 08-251 | Boumerdes | Cow | 21/05/00 | 36°43'35.34"N | 3°42'35.48"E |
| Algeria | 08-252 | Tipaza | Dog | 20/03/00 | 36°34'24.91"N | 2°25'15.83"E |
| Algeria | 08-253 | Tizi-Ouzou | Dog | 5/11/00 | 36°33'.21"N | 4°5'.22"E |
| Algeria | 08-254 | Boumerdes | Cat | 13/04/00 | 36°43'35.34"N | 3°42'35.48"E |
| Algeria | 08-256 | Alger | Dog | 26/01/00 | 36°46'31.80"N | 3° 3'34.92"E |
| Algeria | 08-257 | Tipaza | Dog | 6/07/00 | 36°34'24.91"N | 2°25'15.83"E |
| Algeria | 08-258 | Alger | Dog | 7/02/00 | 36°46'31.80"N | 3° 3'34.92"E |
| Algeria | 08-259 | Bouira | Sheep | 25/06/00 | 36°12'30.32"N | 3°55'30.18"E |
| Algeria | 08-260 | Blida | Dog | 12/11/00 | 36°28'22.44"N | 2°49'30.36"E |
| Tunisia | 86075TUN | NO DATA | Human | 1986 |  |  |
| Tunisia | 86127TUN | NO DATA | Human | 1986 |  |  |
| Tunisia | 86128TUN | NO DATA | Human | 1986 |  |  |
| Tunisia | 86130TUN | NO DATA | Human | 1986 |  |  |
| Tunisia | 86131TUN | NO DATA | Human | 1986 |  |  |
| Tunisia | 8727TUN | NO DATA | Human | 1986 |  |  |
| Tunisia | 8676TUN | NO DATA | Human | 1986 |  |  |
| Spain | 09-007 | Melilla | Dog | 2005 | 35°17'30.97"N | 2°56'18.54"W |
| Spain | 09-008 | Melilla | Dog | 2002 | 35°17'30.97"N | 2°56'18.54"W |
| Spain | 09-009 | Melilla | Dog | 1995 | 35°17'30.97"N | 2°56'18.54"W |
| Spain | 09-010 | Melilla | Cat | 1987 | 35°17'30.97"N | 2°56'18.54"W |
| Spain | 09-011 | Ceuta | Dog | 1991 | 35°53'17.83"N | 5°18'58.30"W |
| Spain | 09-012 | Ceuta | Dog | 2008 | 35°53'17.83"N | 5°18'58.30"W |
| Spain | 09-013 | Melilla | Dog | 1993 | 35°17'30.97"N | 2°56'18.54"W |
| Spain | 09-014 | Melilla | Dog | 1992 | 35°17'30.97"N | 2°56'18.54"W |
| Spain | 09-015 | Melilla | Dog | 1991 | 35°17'30.97"N | 2°56'18.54"W |

**Table S2**. Precise dates (month) of sampling and precise geographical locality (city) for isolates used in the spatio-temporal analysis

| **Countries** | **Isolates** | **Cities** | **Species** | **Dates** | **Latitude** | **Longitude** |
| --- | --- | --- | --- | --- | --- | --- |
| Algeria | 08-121 | Alger | Dog | 10/01/07 | 36°46'31.80"N | 3° 3'34.92"E |
| Algeria | 08-122 | Blida | Dog | 10/01/07 | 36°28'22.44"N | 2°49'30.36"E |
| Algeria | 08-123 | blida | Jackal | 13/01/07 | 36°28'22.44"N | 2°49'30.36"E |
| Algeria | 08-124 | Ain Defla | Dog | 10/02/07 | 36°4'2.36"N | 4°33'19.09"E |
| Algeria | 08-125 | Bejaia | Dog | 13/02/07 | 36°32'34.05"N | 4°58'26.17"E |
| Algeria | 08-127 | Msila | Dog | 4/03/07 | 35°7'8.31"N | 4°25'44.65"E |
| Algeria | 08-128 | Bouira | Dog | 16/03/07 | 36°12'30.32"N | 3°55'30.18"E |
| Algeria | 08-129 | Msila | Dog | 23/03/07 | 35°7'8.31"N | 4°25'44.65"E |
| Algeria | 08-130 | Tipaza | Dog | 2/04/07 | 36°34'24.91"N | 2°25'15.83"E |
| Algeria | 08-131 | Bejaia | Dog | 17/04/07 | 36°32'34.05"N | 4°58'26.17"E |
| Algeria | 08-132 | Biskra | Dog | 28/04/07 | 34°51'20.16"N | 5°43'41.16"E |
| Algeria | 08-133 | Alger | Dog | 28/04/07 | 36°46'31.80"N | 3° 3'34.92"E |
| Algeria | 08-134 | Bejaia | Dog | 28/04/07 | 36°32'34.05"N | 4°58'26.17"E |
| Algeria | 08-135 | Blida | Sheep | 12/05/07 | 36°28'22.44"N | 2°49'30.36"E |
| Algeria | 08-136 | Djelfa | Donkey | 23/05/07 | 34°40'32.52"N | 3°15'7.20"E |
| Algeria | 08-137 | Bouira | Dog | 29/05/07 | 36°12'30.32"N | 3°55'30.18"E |
| Algeria | 08-139 | Tissemsilt | Dog | 2/06/07 | 35°45'9.94"N | 1°52'2.06"E |
| Algeria | 08-140 | Bouira | Dog | 4/06/07 | 36°12'30.32"N | 3°55'30.18"E |
| Algeria | 08-141 | Chlef | Cow | 9/06/07 | 36°9'54.36"N | 1°19'37.20"E |
| Algeria | 08-142 | Tipaza | Dog | 11/06/07 | 36°34'24.91"N | 2°25'15.83"E |
| Algeria | 08-143 | Boumerdes | Dog | 11/06/07 | 36°43'35.34"N | 3°42'35.48"E |
| Algeria | 08-144 | Relizane | Donkey | 17/06/07 | 35°44'37.68"N | 0°33'29.88"E |
| Algeria | 08-145 | chlef | Cow | 19/06/07 | 36°9'54.36"N | 1°19'37.20"E |
| Algeria | 08-146 | Médéa | Cat | 23/06/07 | 36°15'46.80"N | 2°45'29.52"E |
| Algeria | 08-147 | Borj Bou arreridj | Goat | 18/04/04 | 35°59'.35"N | 4°58'.1"W |
| Algeria | 08-149 | Médéa | Dog | 14/05/04 | 36°15'46.80"N | 2°45'29.52"E |
| Algeria | 08-150 | Ouargla | Dog | 30/11/05 | 31°56'44.52"N | 5°19'31.44"E |
| Algeria | 08-151 | Tizi-Ouzou | Dog | 27/06/07 | 36°33'.21"N | 4°5'.22"E |
| Algeria | 08-152 | Msila | Dog | 4/07/07 | 35°7'8.31"N | 4°25'44.65"E |
| Algeria | 08-153 | Constantine | Dog | 9/07/07 | 36°21'46.44"N | 6°36'31.32"E |
| Algeria | 08-154 | Blida | Jackal | 20/05/04 | 36°15'46.80"N | 2°45'29.52"E |
| Algeria | 08-155 | sétif | Dog | 10/07/07 | 36°11'18.96"N | 5°24'25.92"E |
| Algeria | 08-156 | Tissemsilt | Dog | 14/02/05 | 35°45'9.94"N | 1°52'2.06"E |
| Algeria | 08-157 | Alger | Dog | 18/07/07 | 36°46'31.80"N | 3° 3'34.92"E |
| Algeria | 08-159 | Boumerdes | Sheep | 24/07/07 | 36°43'35.34"N | 3°42'35.48"E |
| Algeria | 08-161 | Tipaza | Dog | 31/07/07 | 36°34'24.91"N | 2°25'15.83"E |
| Algeria | 08-162 | Borj Bou arreridj | Bovine | 6/08/07 | 35°59'.35"N | 4°58'.1"W |
| Algeria | 08-165 | Alger | Goat | 16/08/07 | 36°46'31.80"N | 3° 3'34.92"E |
| Algeria | 08-166 | Blida | Dog | 18/08/07 | 36°28'22.44"N | 2°49'30.36"E |
| Algeria | 08-167 | Médéa | Dog | 11/09/07 | 36°15'46.80"N | 2°45'29.52"E |
| Algeria | 08-170 | Chlef | Cat | 30/09/07 | 36°9'54.36"N | 1°19'37.20"E |
| Algeria | 08-172 | Bouira | Dog | 4/11/07 | 36°12'30.32"N | 3°55'30.18"E |
| Algeria | 08-173 | Chlef | Dog | 5/11/07 | 36°9'54.36"N | 1°19'37.20"E |
| Algeria | 08-174 | Djelfa | Dog | 8/12/07 | 34°40'32.52"N | 3°15'7.20"E |
| Algeria | 08-175 | Alger | Cat | 11/12/07 | 36°46'31.80"N | 3° 3'34.92"E |
| Algeria | 08-176 | Msila | Donkey | 22/12/07 | 35°7'8.31"N | 4°25'44.65"E |
| Algeria | 08-177 | Alger | Dog | 25/12/07 | 36°46'31.80"N | 3° 3'34.92"E |
| Algeria | 08-178 | Alger | Dog | 25/12/07 | 36°46'31.80"N | 3° 3'34.92"E |
| Algeria | 08-180 | Alger | Dog | 8/01/08 | 36°46'31.80"N | 3° 3'34.92"E |
| Algeria | 08-181 | Alger | Donkey | 9/01/08 | 36°46'31.80"N | 3° 3'34.92"E |
| Algeria | 08-183 | Médéa | Dog | 22/01/08 | 36°15'46.80"N | 2°45'29.52"E |
| Algeria | 08-185 | Blida | Sheep | 26/01/08 | 36°28'22.44"N | 2°49'30.36"E |
| Algeria | 08-187 | Boumerdes | Dog | 3/02/08 | 36°43'35.34"N | 3°42'35.48"E |
| Algeria | 08-189 | Alger | Dog | 6/02/08 | 36°46'31.80"N | 3° 3'34.92"E |
| Algeria | 08-190 | Bejaia | Sheep | 12/02/08 | 36°32'34.05"N | 4°58'26.17"E |
| Algeria | 08-191 | Ain Defla | Dog | 13/02/08 | 36°4'2.36"N | 4°33'19.09"E |
| Algeria | 08-192 | Msila | Dog | 12/02/08 | 35°7'8.31"N | 4°25'44.65"E |
| Algeria | 08-193 | Alger | Dog | 21/02/08 | 36°46'31.80"N | 3° 3'34.92"E |
| Algeria | 08-194 | Tissemsilt | Dog | 25/02/08 | 35°45'9.94"N | 1°52'2.06"E |
| Algeria | 08-195 | Alger | Dog | 26/02/08 | 36°46'31.80"N | 3° 3'34.92"E |
| Algeria | 08-196 | Blida | Cat | 29/02/08 | 36°28'22.44"N | 2°49'30.36"E |
| Algeria | 08-197 | Boumerdes | Cat | 3/03/08 | 36°43'35.34"N | 3°42'35.48"E |
| Algeria | 08-198 | Alger | Dog | 5/03/08 | 36°46'31.80"N | 3° 3'34.92"E |
| Algeria | 08-199 | Ain Defla | caprine | 13/03/08 | 36°4'2.36"N | 4°33'19.09"E |
| Algeria | 08-202 | Alger | Dog | 17/03/08 | 36°46'31.80"N | 3° 3'34.92"E |
| Algeria | 08-203 | Tipaza | Dog | 18/03/08 | 36°34'24.91"N | 2°25'15.83"E |
| Algeria | 08-204 | Alger | Dog | 29/03/08 | 36°46'31.80"N | 3° 3'34.92"E |
| Algeria | 08-205 | Tipaza | Cow | 3/04/08 | 36°34'24.91"N | 2°25'15.83"E |
| Algeria | 08-207 | Alger | Dog | 10/04/08 | 36°46'31.80"N | 3° 3'34.92"E |
| Algeria | 08-208 | Tipaza | Dog | 12/04/08 | 36°34'24.91"N | 2°25'15.83"E |
| Algeria | 08-209 | Alger | Cat | 23/12/01 | 36°46'31.80"N | 3° 3'34.92"E |
| Algeria | 08-210 | Alger | Dog | 1/12/01 | 36°46'31.80"N | 3° 3'34.92"E |
| Algeria | 08-211 | Blida | Dog | 20/12/01 | 36°28'22.44"N | 2°49'30.36"E |
| Algeria | 08-212 | Médéa | Cow | 16/07/01 | 36°15'46.80"N | 2°45'29.52"E |
| Algeria | 08-214 | Bejaia | Dog | 25/11/01 | 36°32'34.05"N | 4°58'26.17"E |
| Algeria | 08-215 | Blida | Cow | 23/11/01 | 36°28'22.44"N | 2°49'30.36"E |
| Algeria | 08-216 | Tizi-Ouzou | Dog | 13/05/01 | 36°33'.21"N | 4°5'.22"E |
| Algeria | 08-217 | Tipaza | Dog | 18/04/01 | 36°34'24.91"N | 2°25'15.83"E |
| Algeria | 08-218 | Boumerdes | Cat | 6/11/01 | 36°43'35.34"N | 3°42'35.48"E |
| Algeria | 08-219 | Blida | Cow | 29/10/01 | 36°28'22.44"N | 2°49'30.36"E |
| Algeria | 08-220 | Tizi-Ouzou | Cow | 23/05/01 | 36°33'.21"N | 4°5'.22"E |
| Algeria | 08-221 | Borj Bou arreridj | Dog | 2/03/02 | 35°59'.35"N | 4°58'.1"W |
| Algeria | 08-222 | Boumerdes | Dog | 22/05/02 | 36°43'35.34"N | 3°42'35.48"E |
| Algeria | 08-223 | Médéa | Dog | 13/06/02 | 36°15'46.80"N | 2°45'29.52"E |
| Algeria | 08-224 | Alger | Dog | 23/09/02 | 36°46'31.80"N | 3° 3'34.92"E |
| Algeria | 08-225 | Boumerdes | Dog | 29/09/02 | 36°43'35.34"N | 3°42'35.48"E |
| Algeria | 08-226 | Tipaza | Dog | 22/04/02 | 36°34'24.91"N | 2°25'15.83"E |
| Algeria | 08-227 | Blida | Dog | 1/06/02 | 36°28'22.44"N | 2°49'30.36"E |
| Algeria | 08-228 | Msila | Sheep | 15/05/02 | 35°7'8.31"N | 4°25'44.65"E |
| Algeria | 08-229 | Boumerdes | Dog | 6/04/02 | 36°28'22.44"N | 2°49'30.36"E |
| Algeria | 08-230 | Batna | Cow | 4/02/02 | 35°33'.19"N | 6°10'.42"E |
| Algeria | 08-231 | Tipaza | Dog | 27/02/02 | 36°34'24.91"N | 2°25'15.83"E |
| Algeria | 08-233 | Médéa | Jackal | 26/02/03 | 36°15'46.80"N | 2°45'29.52"E |
| Algeria | 08-234 | Blida | Goat | 14/07/03 | 36°28'22.44"N | 2°49'30.36"E |
| Algeria | 08-235 | Alger | Cat | 25/05/03 | 36°46'31.80"N | 3° 3'34.92"E |
| Algeria | 08-236 | Tipaza | Dog | 16/04/03 | 36°34'24.91"N | 2°25'15.83"E |
| Algeria | 08-237 | Bouira | Dog | 2/03/03 | 36°12'30.32"N | 3°55'30.18"E |
| Algeria | 08-239 | Médéa | Sheep | 26/03/03 | 36°15'46.80"N | 2°45'29.52"E |
| Algeria | 08-240 | Boumerdes | Sheep | 30/03/03 | 36°43'35.34"N | 3°42'35.48"E |
| Algeria | 08-241 | Alger | Cat | 17/08/03 | 36°46'31.80"N | 3° 3'34.92"E |
| Algeria | 08-242 | Alger | Dog | 10/05/03 | 36°46'31.80"N | 3° 3'34.92"E |
| Algeria | 08-243 | Relizane | Calf | 26/03/03 | 35°44'37.68"N | 0°33'29.88"E |
| Algeria | 08-245 | Blida | Calf | 2/04/03 | 36°28'22.44"N | 2°49'30.36"E |
| Algeria | 08-246 | Chlef | Dog | 12/05/03 | 36°9'54.36"N | 1°19'37.20"E |
| Algeria | 08-247 | Blida | Dog | 19/02/03 | 36°28'22.44"N | 2°49'30.36"E |
| Algeria | 08-248 | Alger | Dog | 1/12/04 | 36°46'31.80"N | 3° 3'34.92"E |
| Algeria | 08-249 | Alger | Dog | 15/05/04 | 36°46'31.80"N | 3° 3'34.92"E |
| Algeria | 08-250 | Tipaza | Bovine | 18/08/04 | 36°34'24.91"N | 2°25'15.83"E |
| Algeria | 08-251 | Boumerdes | Cow | 22/05/04 | 36°43'35.34"N | 3°42'35.48"E |
| Algeria | 08-252 | Tipaza | Dog | 21/03/04 | 36°34'24.91"N | 2°25'15.83"E |
| Algeria | 08-253 | Tizi-Ouzou | Dog | 6/11/04 | 36°33'.21"N | 4°5'.22"E |
| Algeria | 08-254 | Boumerdes | Cat | 14/04/04 | 36°43'35.34"N | 3°42'35.48"E |
| Algeria | 08-256 | Alger | Dog | 27/01/04 | 36°46'31.80"N | 3° 3'34.92"E |
| Algeria | 08-257 | Tipaza | Dog | 7/07/04 | 36°34'24.91"N | 2°25'15.83"E |
| Algeria | 08-258 | Alger | Dog | 8/02/04 | 36°46'31.80"N | 3° 3'34.92"E |
| Algeria | 08-259 | Bouira | Sheep | 26/06/04 | 36°12'30.32"N | 3°55'30.18"E |
| Algeria | 08-260 | Blida | Dog | 13/11/04 | 36°28'22.44"N | 2°49'30.36"E |
| Morocco | 08-001 | Chichaoua | Dog | 9/01/07 | 31°32'47.40"N | 8°45'45.36"W |
| Morocco | 08-002 | Azilal | caprine | 19/01/07 | 31°58'0.84"N | 6°33'58.32"W |
| Morocco | 08-003 | Marrakech | Dog | 26/01/07 | 31°38'21.01"N | 8° 0'17.17"W |
| Morocco | 08-004 | Ouarzazate | Equine | 29/01/07 | 30°55'8.00"N | 6°54'13.75"W |
| Morocco | 08-005 | Marrakech | Equine | 16/02/07 | 31°38'21.01"N | 8° 0'17.17"W |
| Morocco | 08-006 | Ouarzazate | Dog | 22/02/07 | 30°55'8.00"N | 6°54'13.75"W |
| Morocco | 08-007 | Ouarzazate | Dog | 22/02/07 | 30°55'8.00"N | 6°54'13.75"W |
| Morocco | 08-008 | Ouarzazate | Equine | 22/02/07 | 30°55'8.00"N | 6°54'13.75"W |
| Morocco | 08-009 | Chichaoua | Bovine | 23/02/07 | 31°32'47.40"N | 8°45'45.36"W |
| Morocco | 08-010 | Marrakech | Bovine | 12/03/07 | 31°38'21.01"N | 8° 0'17.17"W |
| Morocco | 08-011 | Marrakech | Dog | 29/03/07 | 31°38'21.01"N | 8° 0'17.17"W |
| Morocco | 08-012 | Ouarzazate | Equine | 3/04/07 | 30°55'8.00"N | 6°54'13.75"W |
| Morocco | 08-013 | Marrakech | Dog | 9/04/07 | 31°38'21.01"N | 8° 0'17.17"W |
| Morocco | 08-014 | Marrakech | Donkey | 14/05/07 | 31°38'21.01"N | 8° 0'17.17"W |
| Morocco | 08-015 | Marrakech | Dog | 1/06/07 | 31°38'21.01"N | 8° 0'17.17"W |
| Morocco | 08-016 | Marrakech | Dog | 6/06/07 | 31°38'21.01"N | 8° 0'17.17"W |
| Morocco | 08-017 | Marrakech | Dog | 4/07/07 | 31°38'21.01"N | 8° 0'17.17"W |
| Morocco | 08-018 | Beni mellal | Donkey | 24/07/07 | 32°20'8.81"N | 6°21'21.56"W |
| Morocco | 08-019 | Marrakech | Dog | 14/09/07 | 31°38'21.01"N | 8° 0'17.17"W |
| Morocco | 08-020 | Marrakech | Donkey | 6/10/07 | 31°38'21.01"N | 8° 0'17.17"W |
| Morocco | 08-021 | Marrakech | Dog | 31/10/07 | 31°38'21.01"N | 8° 0'17.17"W |
| Morocco | 08-022 | Azilal | Bovine | 2/11/07 | 31°58'0.84"N | 6°33'58.32"W |
| Morocco | 08-023 | Marrakech | Dog | 7/11/07 | 31°38'21.01"N | 8° 0'17.17"W |
| Morocco | 08-024 | Ouarzazate | Sheep | 20/11/07 | 30°55'8.00"N | 6°54'13.75"W |
| Morocco | 08-025 | Tanger | Bovine | 23/02/04 | 35°46'50.52"N | 5°48'41.04"W |
| Morocco | 08-031 | Sidi kacem | Dog | 15/10/04 | 33°16'60.00"N | 7°52'0.00"W |
| Morocco | 08-033 | Kenitra | Dog | 11/11/04 | 34°16'22.44"N | 6°34'33.60"W |
| Morocco | 08-034 | Tanger | Dog | 22/11/04 | 35°46'50.52"N | 5°48'41.04"W |
| Morocco | 08-035 | Kenitra | Bovine | 23/11/04 | 34°16'22.44"N | 6°34'33.60"W |
| Morocco | 08-038 | Sidi kacem | Bovine | 8/04/05 | 33°16'60.00"N | 7°52'0.00"W |
| Morocco | 08-039 | Kenitra | Bovine | 8/04/05 | 34°16'22.44"N | 6°34'33.60"W |
| Morocco | 08-040 | Casa | Equine | 2/10/06 | 33°35'20.04"N | 7°36'32.04"W |
| Morocco | 08-041 | Benslimane | Donkey | 24/11/06 | 33°36'35.28"N | 7° 7'16.32"W |
| Morocco | 08-043 | Casa | Dog | 22/01/07 | 33°35'20.04"N | 7°36'32.04"W |
| Morocco | 08-045 | Benslimane | Dog | 20/02/07 | 33°36'35.28"N | 7° 7'16.32"W |
| Morocco | 08-046 | Benslimane | Bovine | 20/02/07 | 33°36'35.28"N | 7° 7'16.32"W |
| Morocco | 08-047 | Casa | Dog | 26/02/07 | 33°35'20.04"N | 7°36'32.04"W |
| Morocco | 08-048 | Settat | Dog | 26/03/07 | 33°0'22.32"N | 7°37'9.48"W |
| Morocco | 08-049 | Benslimane | Bovine | 5/04/07 | 33°36'35.28"N | 7° 7'16.32"W |
| Morocco | 08-050 | Settat | Dog | 10/04/07 | 33°0'22.32"N | 7°37'9.48"W |
| Morocco | 08-051 | Benslimane | Dog | 11/04/07 | 33°36'35.28"N | 7° 7'16.32"W |
| Morocco | 08-052 | Khouribga | Dog | 25/04/07 | 32°52'51.60"N | 6°54'39.96"W |
| Morocco | 08-053 | Benslimane | Bovine | 2/05/07 | 33°36'35.28"N | 7° 7'16.32"W |
| Morocco | 08-054 | Casa | Dog | 7/05/07 | 33°35'20.04"N | 7°36'32.04"W |
| Morocco | 08-055 | Casa | Dog | 11/05/07 | 33°35'20.04"N | 7°36'32.04"W |
| Morocco | 08-056 | Settat | Dog | 28/07/05 | 33°0'22.32"N | 7°37'9.48"W |
| Morocco | 08-057 | Settat | Sheep | 21/06/07 | 33°0'22.32"N | 7°37'9.48"W |
| Morocco | 08-058 | Casa | Dog | 12/07/07 | 33°35'20.04"N | 7°36'32.04"W |
| Morocco | 08-059 | Nador | Dog | 5/01/07 | 33°37'0.00"N | 3°44'0.00"W |
| Morocco | 08-060 | Berkane | Bovine | 5/01/07 | 34°55'28.56"N | 2°20'2.40"W |
| Morocco | 08-061 | Berkane | Donkey | 2/02/07 | 34°55'28.56"N | 2°20'2.40"W |
| Morocco | 08-062 | Nador | Dog | 9/02/07 | 33°37'0.00"N | 3°44'0.00"W |
| Morocco | 08-063 | Oujda | Dog | 22/02/07 | 34°41'39.48"N | 1°55'5.88"W |
| Morocco | 08-064 | Berkane | Dog | 26/02/07 | 34°55'28.56"N | 2°20'2.40"W |
| Morocco | 08-065 | Al hoceima | Dog | 13/03/07 | 35°14'57.48"N | 3°56'13.60"W |
| Morocco | 08-066 | Jerrada | Dog | 14/03/07 | 34°18'36.00"N | 2° 9'38.88"W |
| Morocco | 08-067 | Taourirt | Donkey | 23/04/07 | 32°41'57.01"N | 9° 4'28.79"W |
| Morocco | 08-068 | Berkane | Dog | 21/05/07 | 34°55'28.56"N | 2°20'2.40"W |
| Morocco | 08-069 | Berkane | Donkey | 31/05/07 | 34°55'28.56"N | 2°20'2.40"W |
| Morocco | 08-070 | Oujda | Dog | 18/06/07 | 34°41'39.48"N | 1°55'5.88"W |
| Morocco | 08-071 | Berkane | Dog | 28/06/07 | 34°55'28.56"N | 2°20'2.40"W |
| Morocco | 08-072 | Berkane | Bovine | 10/09/07 | 34°55'28.56"N | 2°20'2.40"W |
| Morocco | 08-073 | Taourirt | Dog | 3/08/07 | 32°41'57.01"N | 9° 4'28.79"W |
| Morocco | 08-074 | Berkane | Bovine | 9/08/07 | 34°55'28.56"N | 2°20'2.40"W |
| Morocco | 08-075 | Taourirt | Donkey | 13/08/07 | 32°41'57.01"N | 9° 4'28.79"W |
| Morocco | 08-076 | Taourirt | Equine | 13/08/07 | 32°41'57.01"N | 9° 4'28.79"W |
| Morocco | 08-077 | Oujda | Sheep | 27/08/07 | 34°41'39.48"N | 1°55'5.88"W |
| Morocco | 08-078 | Oujda | caprine | 15/11/07 | 34°41'39.48"N | 1°55'5.88"W |
| Morocco | 08-079 | Rabat | Equine | 3/01/07 | 33°59'25.80"N | 6°52'13.44"W |
| Morocco | 08-080 | Rabat | Dog | 4/05/07 | 33°59'25.80"N | 6°52'13.44"W |
| Morocco | 08-081 | Rabat | Dog | 7/05/07 | 33°59'25.80"N | 6°52'13.44"W |
| Morocco | 08-082 | Rabat | Dog | 28/11/06 | 33°59'25.80"N | 6°52'13.44"W |
| Morocco | 08-083 | Rabat | Dog | 21/12/06 | 33°59'25.80"N | 6°52'13.44"W |
| Morocco | 08-084 | Rabat | Dog | 27/07/07 | 33°59'25.80"N | 6°52'13.44"W |
| Morocco | 08-085 | Rabat | Dog | 6/07/07 | 33°59'25.80"N | 6°52'13.44"W |
| Morocco | 08-086 | Sidi kacem | Dog | 22/01/07 | 33°16'60.00"N | 7°52'0.00"W |
| Morocco | 08-087 | Sidi kacem | Dog | 31/01/07 | 33°16'60.00"N | 7°52'0.00"W |
| Morocco | 08-089 | Errachidia | Dog | 3/04/07 | 31°56'41.06"N | 4°24'2.16"W |
| Morocco | 08-090 | El hajeb | Bovine | 9/04/07 | 33°41'53.16"N | 5°21'53.23"W |
| Morocco | 08-091 | Errachidia | Dog | 10/04/07 | 31°56'41.06"N | 4°24'2.16"W |
| Morocco | 08-092 | Figuig | Mule | 10/04/07 | 32°6'3.60"N | 1°13'17.04"W |
| Morocco | 08-093 | Taza | Bovine | 10/04/07 | 34°13'58.79"N | 3°56'57.03"W |
| Morocco | 08-094 | Khenifra | Bovine | 12/04/07 | 32°56'18.96"N | 5°40'5.52"W |
| Morocco | 08-095 | Sidi kacem | Dog | 13/04/07 | 33°16'60.00"N | 7°52'0.00"W |
| Morocco | 08-096 | Sidi kacem | Bovine | 24/04/07 | 33°16'60.00"N | 7°52'0.00"W |
| Morocco | 08-097 | Taza | Dog | 24/04/07 | 34°13'58.79"N | 3°56'57.03"W |
| Morocco | 08-098 | Sidi kacem | Bovine | 7/05/07 | 33°16'60.00"N | 7°52'0.00"W |
| Morocco | 08-099 | Ifrane | Bovine | 9/05/07 | 34°2'60.00"N | 3°46'0.00"W |
| Morocco | 08-100 | Errachidia | Dog | 21/05/07 | 31°56'41.06"N | 4°24'2.16"W |
| Morocco | 08-101 | Sidi kacem | Bovine | 22/06/07 | 33°16'60.00"N | 7°52'0.00"W |
| Morocco | 08-102 | Errachidia | Dog | 27/06/07 | 31°56'41.06"N | 4°24'2.16"W |
| Morocco | 08-103 | Fès | Bovine | 6/07/07 | 34°2'12.70"N | 5° 0'10.01"W |
| Morocco | 08-104 | Figuig | Sheep | 10/07/07 | 32°6'3.60"N | 1°13'17.04"W |
| Morocco | 08-105 | El hajeb | Dog | 12/07/07 | 33°41'53.16"N | 5°21'53.23"W |
| Morocco | 08-106 | Taounate | Bovine | 23/07/07 | 34°32'8.88"N | 4°38'24.36"W |
| Morocco | 08-110 | Khenifra | Mule | 3/10/07 | 32°56'18.96"N | 5°40'5.52"W |
| Morocco | 08-111 | Agadir | Dog | 21/02/07 | 30° 9'0.00"N | 8°58'0.00"W |
| Morocco | 08-112 | Agadir | Bovine | 2/04/07 | 30° 9'0.00"N | 8°58'0.00"W |
| Morocco | 08-113 | Agadir | Cat | 12/07/07 | 30° 9'0.00"N | 8°58'0.00"W |
| Morocco | 08-114 | Agadir | Dog | 22/05/06 | 30° 9'0.00"N | 8°58'0.00"W |
| Morocco | 08-116 | Agadir | Dog | 15/08/06 | 30° 9'0.00"N | 8°58'0.00"W |
| Morocco | 08-117 | Agadir | Dog | 28/03/07 | 30° 9'0.00"N | 8°58'0.00"W |
| Morocco | 08-118 | Agadir | Dog | 30/06/06 | 30° 9'0.00"N | 8°58'0.00"W |
| Morocco | 08-119 | Agadir | Dog | 21/12/04 | 30° 9'0.00"N | 8°58'0.00"W |
| Morocco | 08-297 | Sidi kacem | Bovine | 16/03/05 | 33°16'60.00"N | 7°52'0.00"W |
| Morocco | 08-298 | Tanger | Bovine | 27/04/04 | 35°46'50.52"N | 5°48'41.04"W |
| Morocco | 08-299 | Tanger | Bovine | 14/07/04 | 35°46'50.52"N | 5°48'41.04"W |
| Morocco | 08-302 | Tanger | Bovine | 7/01/05 | 35°46'50.52"N | 5°48'41.04"W |
| Morocco | 08-303 | Tanger | Bovine | 28/07/04 | 35°46'50.52"N | 5°48'41.04"W |
| Morocco | 08-304 | Tanger | Bovine | 3/08/04 | 35°46'50.52"N | 5°48'41.04"W |
| Morocco | 08-305 | Tanger | Dog | 12/08/04 | 35°46'50.52"N | 5°48'41.04"W |
| Morocco | 08-306 | Tanger | Bovine | 27/02/07 | 35°46'50.52"N | 5°48'41.04"W |
| Morocco | 08-307 | Tanger | Bovine | 14/07/04 | 35°46'50.52"N | 5°48'41.04"W |
| Morocco | 08-311 | Benslimane | Bovine | 1-mai | 33°36'35.28"N | 7° 7'16.32"W |
| Morocco | 08-312 | Benslimane | Dog | 1/05/07 | 33°36'35.28"N | 7° 7'16.32"W |
| Morocco | 08-314 | Casa | Dog | 1/03/08 | 33°35'20.04"N | 7°36'32.04"W |
| Morocco | 08-316 | Casa | Bovine | 1/03/08 | 33°35'20.04"N | 7°36'32.04"W |
| Morocco | 08-317 | Benslimane | Dog | 1/04/08 | 33°36'35.28"N | 7° 7'16.32"W |
| Morocco | 08-318 | Oujda | Dog | 22/02/08 | 34°41'39.48"N | 1°55'5.88"W |
| Morocco | 08-319 | Al hoceima | Dog | 16/04/08 | 35°14'57.48"N | 3°56'13.60"W |
| Morocco | 08-320 | Berkane | Dog | 29/04/08 | 34°55'28.56"N | 2°20'2.40"W |
| Morocco | 08-322 | Marrakech | Dog | 1/02/07 | 31°38'21.01"N | 8° 0'17.17"W |
| Morocco | 08-323 | Ouarzazate | Dog | 1/02/07 | 30°55'8.00"N | 6°54'13.75"W |
| Morocco | 08-324 | Marrakech | Dog | 1/02/07 | 31°38'21.01"N | 8° 0'17.17"W |
| Morocco | 08-325 | Chichaoua | Bovine | 1/03/2007 | 31°32'47.40"N | 8°45'45.36"W |
| Morocco | 08-326 | Marrakech | Dog | 1/06/07 | 31°38'21.01"N | 8° 0'17.17"W |
| Morocco | 08-327 | Marrakech | Dog | 1/04/07 | 31°38'21.01"N | 8° 0'17.17"W |
| Morocco | 08-328 | Marrakech | Dog | 1/05/07 | 31°38'21.01"N | 8° 0'17.17"W |
| Morocco | 08-329 | Beni mellal | Donkey | 1/05/07 | 32°20'8.81"N | 6°21'21.56"W |
| Morocco | 08-330 | Marrakech | Dog | 1/10/07 | 31°38'21.01"N | 8° 0'17.17"W |
| Morocco | 08-333 | Beni mellal | Bovine | 1/04/07 | 32°20'8.81"N | 6°21'21.56"W |
| Morocco | 08-334 | Marrakech | Donkey | 1/06/07 | 31°38'21.01"N | 8° 0'17.17"W |
| Morocco | 08-335 | Marrakech | Dog | 1/06/07 | 31°38'21.01"N | 8° 0'17.17"W |

**Table S3**: Human population size data used in this study. Population size estimates were obtained from http://en.wikipedia.org/ and http://www.mongabay.com.

| **Countries** | **Location** | **Latitude** | **Longitude** | **Population size** |
| --- | --- | --- | --- | --- |
| Algeria | AinDefla | 36,06732222 | 4,555302778 | 41200 |
| Algeria | Alger | 36,7755 | 3,0597 | 3518083 |
| Algeria | Batna | 35,55005278 | 6,166783333 | 317206 |
| Algeria | Bejaia | 36,54279167 | 4,973936111 | 182131 |
| Algeria | Biskra | 34,8556 | 5,7281 | 207987 |
| Algeria | Blida | 36,4729 | 2,8251 | 264598 |
| Algeria | BorjBouarreridj | 36,0701 | 4,7661 | 167230 |
| Algeria | Bouira | 36,20842222 | 3,92505 | 52500 |
| Algeria | Boumerdes | 36,72648333 | 3,709855556 | 28500 |
| Algeria | Chlef | 36,1651 | 1,327 | 235062 |
| Algeria | Constantine | 36,3629 | 6,6087 | 507224 |
| Algeria | Djelfa | 34,6757 | 3,252 | 221231 |
| Algeria | Medea | 36,263 | 2,7582 | 155852 |
| Algeria | Msila | 35,118975 | 4,429069444 | 805519 |
| Algeria | Ouargla | 31,9457 | 5,3254 | 183238 |
| Algeria | Relizane | 35,7438 | 0,5583 | 126794 |
| Algeria | Setif | 36,1886 | 5,4072 | 1311413 |
| Algeria | Tipaza | 36,57358611 | 2,421063889 | 506053 |
| Algeria | Tissemsilt | 35,75276111 | 1,867238889 | 51673 |
| Algeria | Tizi-Ouzou | 36,55005833 | 4,083394444 | 77475 |
| Morocco | Agadir | 30,15 | -8,966666667 | 2005703 |
| Morocco | Alhoceima | 35,2493 | -3,937111111 | 70347 |
| Morocco | Azilal | 31,9669 | -6,5662 | 513999 |
| Morocco | Benimellal | 32,33578056 | -6,355988889 | 196146 |
| Morocco | Benslimane | 33,6098 | -7,1212 | 43572 |
| Morocco | Berkane | 34,9246 | -2,334 | 94816 |
| Morocco | Casa | 33,5889 | -7,6089 | 3462648 |
| Morocco | Chichaoua | 31,5465 | -8,7626 | 15657 |
| Morocco | Elhajeb | 33,6981 | -5,364786111 | 22000 |
| Morocco | Errachidia | 31,94473889 | -4,4006 | 128716 |
| Morocco | Fes | 34,03686111 | -5,002780556 | 1223087 |
| Morocco | Figuig | 32,101 | -1,2214 | 12000 |
| Morocco | Ifrane | 34,05 | -3,766666667 | 127677 |
| Morocco | Jerrada | 34,31 | -2,1608 | 78353 |
| Morocco | Kenitra | 34,2729 | -6,576 | 428181 |
| Morocco | Khenifra | 32,9386 | -5,6682 | 89549 |
| Morocco | Khouribga | 32,881 | -6,9111 | 177402 |
| Morocco | Marrakech | 31,63916944 | -8,004769444 | 969420 |
| Morocco | Nador | 33,61666667 | -3,733333333 | 187703 |
| Morocco | Ouarzazate | 30,91888889 | -6,903819444 | 79600 |
| Morocco | Oujda | 34,6943 | -1,9183 | 456524 |
| Morocco | Rabat | 33,9905 | -6,8704 | 1889635 |
| Morocco | Settat | 33,0062 | -7,6193 | 134465 |
| Morocco | Sidikacem | 33,28333333 | -7,866666667 | 79764 |
| Morocco | Tanger | 35,7807 | -5,8114 | 851321 |
| Morocco | Taounate | 34,5358 | -4,6401 | 36587 |
| Morocco | Taourirt | 32,69916944 | -9,074663889 | 94947 |
| Morocco | Taza | 34,23299722 | -3,949175 | 108463 |

**Figure S1**. Estimated epidemiological linkage among the Algerian and Moroccan sampling locations. Linkages in the migration graph supported by Bayes factors >5 are shown for the separate Algerian and Moroccan analyses. The white-magenta color gradient of the lines relates to relative posterior migration rate expectations.


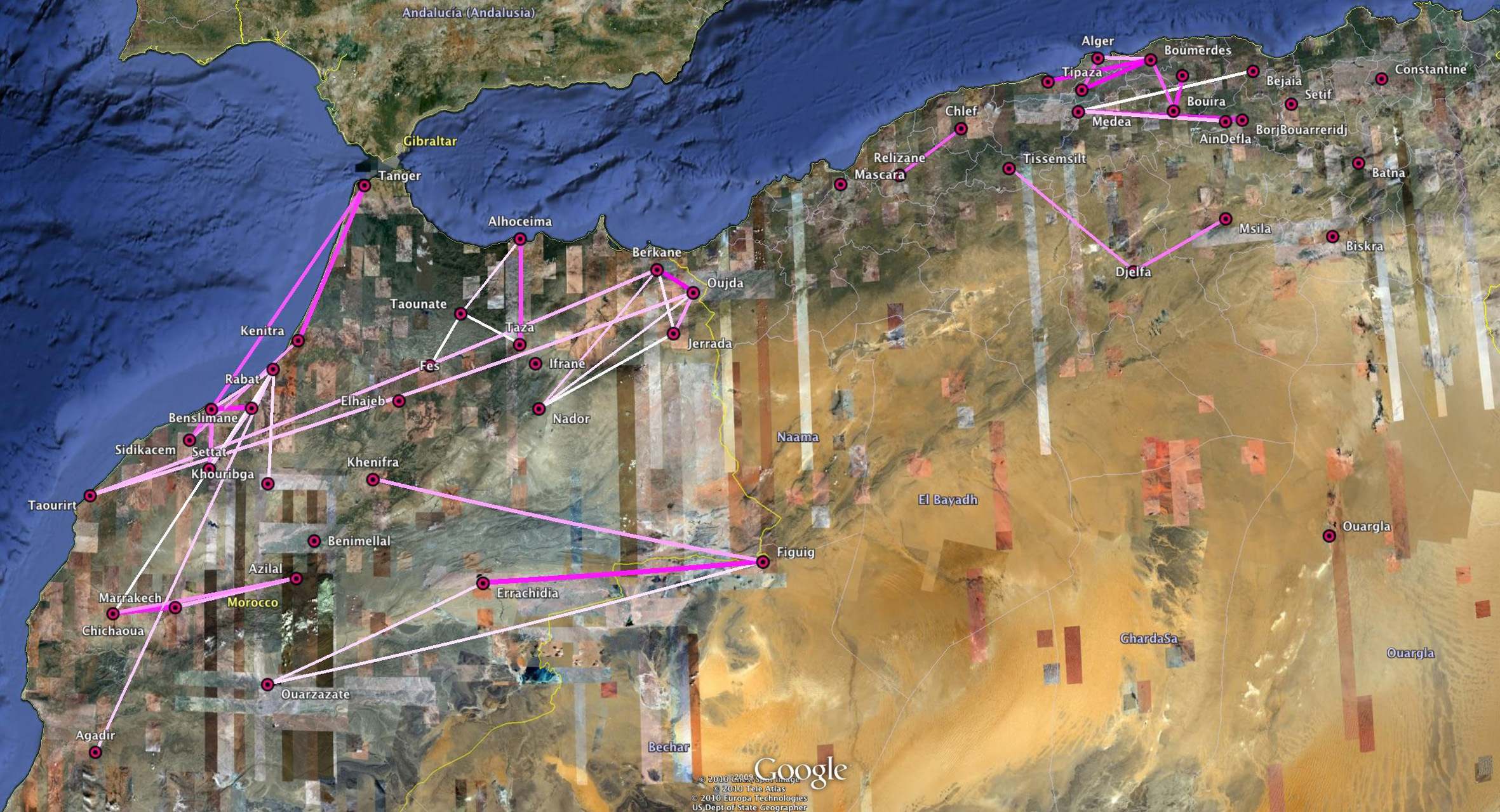


**Figure S2**. Human population size in North Africa per 1x1 km square. The yellow-blue color code indicates values from 0 to 130,000 inhabitants per square km. Data from ref [18].


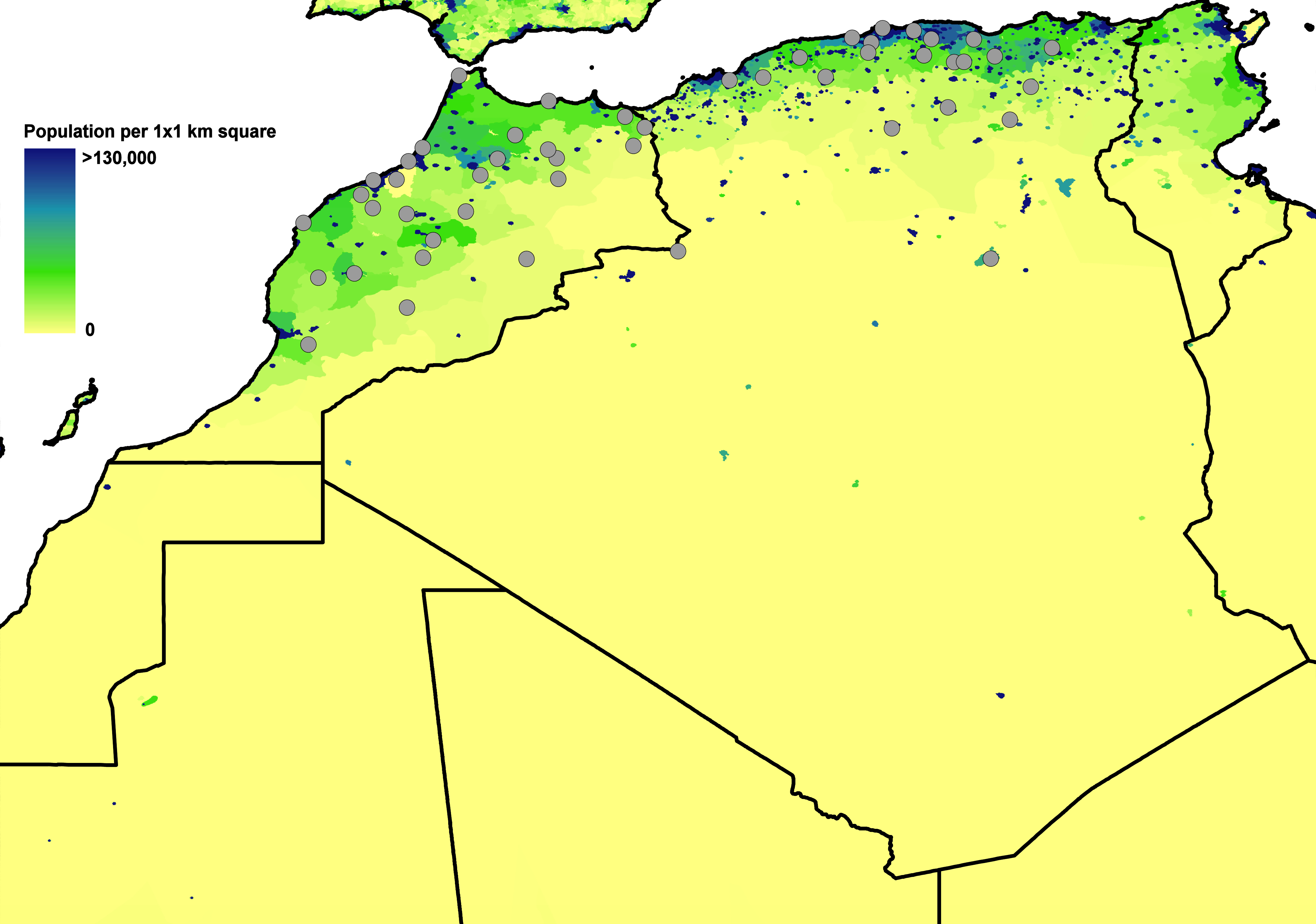

Supplement: Supporting Information S1 — Tables S1, S2, S3 and Figures S1 and S2 (4.34 MB DOC) [file ppat.1001166.s001.doc]
